# Longitudinal Evaluation of Brain Plasticity in Low-Grade Gliomas: fMRI and Graph-Theory Provide Insights on Language Reorganization

**DOI:** 10.3390/cancers15030836

**Published:** 2023-01-29

**Authors:** Luca Pasquini, Kyung K. Peck, Alice Tao, Gino Del Ferraro, Denise D. Correa, Mehrnaz Jenabi, Erik Kobylarz, Zhigang Zhang, Cameron Brennan, Viviane Tabar, Hernán Makse, Andrei I. Holodny

**Affiliations:** 1 Neuroradiology Service, Department of Radiology, Memorial Sloan Kettering Cancer Center, New York, NY 10065, USA; 2 Neuroradiology Unit, NESMOS Department, Sant’Andrea Hospital, La Sapienza University, 00189 Rome, Italy; 3 Department of Medical Physics, Memorial Sloan Kettering Cancer Center, New York, NY 10065, USA; 4 Center for Neural Science, New York University, New York, NY 10003, USA; 5 Department of Neurology, Memorial Sloan Kettering Cancer Center, New York, NY 10065, USA; 6 Department of Neurology and Neuroscience, Weill Medical College of Cornell University, New York, NY 10021, USA; 7 Department of Neurology, Geisel School of Medicine, Dartmouth College, Hanover, NH 03755, USA; 8 Thayer School of Engineering, Dartmouth College, Hanover, NH 03755, USA; 9 Department of Epidemiology and Biostatistics, Memorial Sloan Kettering Cancer Center, New York, NY 10065, USA; 10 Department of Neurosurgery, Memorial Sloan Kettering Cancer Center, New York, NY 10065, USA; 11 Levich Institute and Physics Department, City College of New York, New York, NY 10031, USA; 12 Department of Radiology, Weill Medical College of Cornell University, New York, NY 10065, USA

**Keywords:** glioma, language reorganization, plasticity, fMRI, graph-theory

## Abstract

**Simple Summary:**

This longitudinal study demonstrates the development of language plasticity in right-handed patients undergoing surgery for left-hemispheric low-grade glioma, characterized by the gradually increased involvement of the right hemisphere in language function. Two patterns of language reorganization were identified: type 1 changes may in part be treatment-related; type 2 may be tumor-induced, since atypical language organization was already present at baseline. Increased inter-hemispheric connectivity may represent the initial step in the development of plastic phenomena. This change could be partially compensatory towards clinical deficits and show prognostic value.

**Abstract:**

Language reorganization may represent an adaptive phenomenon to compensate tumor invasion of the dominant hemisphere. However, the functional changes over time underlying language plasticity remain unknown. We evaluated language function in patients with low-grade glioma (LGG), using task-based functional MRI (tb-fMRI), graph-theory and standardized language assessment. We hypothesized that functional networks obtained from tb-fMRI would show connectivity changes over time, with increased right-hemispheric participation. We recruited five right-handed patients (4M, mean age 47.6Y) with left-hemispheric LGG. Tb-fMRI and language assessment were conducted pre-operatively (pre-op), and post-operatively: post-op1 (4–8 months), post-op2 (10–14 months) and post-op3 (16–23 months). We computed the individual functional networks applying optimal percolation thresholding. Language dominance and hemispheric connectivity were quantified by laterality indices (LI) on fMRI maps and connectivity matrices. A fixed linear mixed model was used to assess the intra-patient correlation trend of LI values over time and their correlation with language performance. Individual networks showed increased inter-hemispheric and right-sided connectivity involving language areas homologues. Two patterns of language reorganization emerged: Three/five patients demonstrated a left-to-codominant shift from pre-op to post-op3 (type 1). Two/five patients started as atypical dominant at pre-op, and remained unchanged at post-op3 (type 2). LI obtained from tb-fMRI showed a significant left-to-right trend in all patients across timepoints. There were no significant changes in language performance over time. Type 1 language reorganization may be related to the treatment, while type 2 may be tumor-induced, since it was already present at pre-op. Increased inter-hemispheric and right-side connectivity may represent the initial step to develop functional plasticity.

## 1. Introduction

The majority of right-handed individuals is left-lateralized for language [1]; however, a focal lesion in the dominant hemisphere may lead to the modification of the language dominance [2], which can be observed on functional Magnetic Resonance Imaging (fMRI) [3]. Such phenomenon is known as functional reorganization and may be compensatory in nature, resulting in an increase in the participation of perilesional or distant areas in the function of the affected sites in the brain [4,5].

The functional modifications of brain networks in glioma patients have been described on fMRI [6], especially in low-grade gliomas (LGG) [7], with a progressive fashion over time. Plastic changes may start in the perilesional area, extend to adjacent regions of the same hemisphere (intra-hemispheric reorganization) and finally extend to the right hemisphere (inter-hemispheric reorganization). One of the most important factors of reorganization is considered to be the timing of the insult [7]. LGGs grow slowly, which may allow short- and long-range functional changes to take place and to consolidate over time [8].

Post-operative language reorganization may support the use of multi-step surgery in LGGs [9,10]. Some authors suggested the possibility to enhance plastic changes with targeted therapies to extend surgical radicality [11]. However, the development of language plasticity over time has seldom been explored in patients with brain tumors and the dynamics of functional reorganization are mostly unknown. Therefore, the serial assessment of language organization changes is an important step to better understand the mechanism underlying cortical reorganization. FMRI is an excellent candidate to study functional changes, due to clinical relevance in brain tumor patients [12], wide availability, non-invasiveness, and relatively low cost. Graph-theory has been employed to investigate brain function from a network perspective [13,14]. The application of graph-theory to task-based fMRI can provide useful information about the relationship between active clusters and their relevance in the whole-brain network [15,16].

We aimed to explore the functional reorganization of language over time before and after surgery in a cohort of left-hemispheric LGG patients undergoing surgery, using longitudinal task-based fMRI, graph-theory, and language assessment. We analyzed functional connectivity patterns to explore the network modifications underlying language reorganization. We compared language dominance from conventional fMRI with connectivity information from graph-theory. Our hypotheses were the following: 1) patients with LGGs would show increased activation of right-sided language-related areas over time on fMRI (increased right laterality); 2) graph-theory would demonstrate an increased connectivity of right-sided language-related areas and increased inter-hemispheric participation in language.

## 2. Materials and Methods

### 2.1. Patients

This prospective cohort study was approved by the institutional review board and conducted in agreement with the Helsinki declaration. Written informed consent was obtained from every subject. Patients were recruited with the inclusion criteria: pathologic diagnosis of LGG at first biopsy, right-handedness, newly diagnosed left-hemispheric glioma undergoing surgery, no significant dropout artifacts on any of the scans. Tumor location and eloquent language areas were labeled by a neuroradiologist on MRI. Tumor size was calculated as the product of the two greatest perpendicular diameters on axial fluid attenuated inversion recovery (FLAIR) images. Tumor progression was assessed by a neuroradiologist based on RANO criteria for diffuse LGG [17]. Extent of resection was calculated as (pre-op size)–(post-op 1 size).

Language assessment was performed by a board-certified clinical neuropsychologist prior to tumor resection (pre-op) and at three intervals after surgery: post-op1 (4–8 months), post-op2 (10–14 months), post-op3 (16–23 months). The test battery included: Boston Naming Test (BNT) [18], Phonemic Verbal Fluency (PVF) and Category Fluency (CF) Test [19]. On the BNT, odd and even items were administered at each time point (30/60 items) to minimize practice effects from repeated exposure. Raw test scores were compared with published normative values and converted into z-scores. A z-score ≤ 1.5 standard deviations below the normative mean suggests dysfunction. Handedness was established through the Edinburgh Handedness Inventory [20].

### 2.2. Awake Surgery Mapping

All patients underwent intra-operative, awake speech mapping. A surgical navigation software (BrainLab GmbH, Munich, Germany) was used as per routine, and fMRI data was uploaded to the workstation. The location of Broca’s area (BA) was approximated based on the expected anatomic location, fMRI findings, and the location of the central sulcus. Stimulation of language centers was performed at increasing intensities (in 2 mA peak-to-peak steps) using an Ojemann cortical stimulator and bipolar stimulation probe (trains of 1 msec biphasic pulses at 60 Hertz). The electrocorticogram was simultaneously recorded with an 8-element strip subdural electrode at nearby sites. The presence or absence of speech responses, e.g., hesitation, arrest, paraphasic errors, etc. with counting, picture naming, repetition were documented. The inferior frontal gyrus (IFG) at the operculum was directly stimulated in all patients. Speech arrest was defined as complete interruption of speech with preserved tongue movements, reproducible at least three times and occurring immediately after electrical stimulation of the same cortical area [21]. If speech arrest was not obtained, the stimulated area was expanded to include most of the IFG, up to the middle frontal gyrus and posteriorly towards the sensorimotor strip, depending on the craniotomy.

### 2.3. fMRI Acquisition

Task-based fMRI was acquired at 4 timepoints: before surgical resection (pre-op) for presurgical planning and three times after surgery: post-op1 (4–8 months), post-op2 (10–14 months), post-op3 (16–23 months). Whole-brain MR imaging was performed on 3T scanners using 8 channel head coil. Each patient was scanned with the same scanner over time. The imaging protocol included: functional images acquired with gradient echo-planar imaging (RT = 4000 ms; ET = 40/30 ms; 128 × 128 matrix; 240 mm FOV; 4.5 mm thickness), T1-weighted (RT = 600 ms; ET = 8 ms; 4.5 mm thickness) and T2-weighted (RT = 4000 ms; ET = 102 ms; 4.5 mm thickness) spin-echo axial images, 3D T1-weighted images (RT = 22 ms; ET = 4 ms; 256 × 256 matrix; 1.5 mm thickness), FLAIR images (RT = 10,000 ms; ET = 106 ms; inversion time = 220 ms; 90°flip angle; 256 × 256 matrix; 4.5 mm thickness). To minimize movement, each patient’s head was immobilized in the head coil with a combination of pillow, head straps, and foam rubber pads. During the fMRI acquisition, all patients performed a phonemic verbal fluency task, where they silently generated words that began with specific letters. Each letter was presented visually on a screen as a prompt to generate the words. This task was chosen because it has been the most reliable to assess language laterality in our experience. The task was presented as a block paradigm consisting of 20 s of activation and 40 s of rest. During rest periods, the patient fixated a crosshair image. The patient’s brain activity and head motion were monitored in real-time using dedicated software (Brainwave, Medical Numerics).

### 2.4. fMRI Analysis

Image preprocessing and analysis were performed using Analysis of Functional NeuroImages (AFNI) [22]. Head motion correction was performed using 3D rigid-body registration. Spatial smoothing using a gaussian kernel with full-width-at-half-maximum of 4 mm was applied to improve the signal-to-noise ratio. Removal of linear trend due to signal drift and high frequency noise was performed. Cross-correlation was applied to analyze block-designed paradigms and to generate statistical parametric maps [23]. Modeled waveform corresponding to the task performance block was cross-correlated with all pixel time courses on a pixel-by-pixel basis to identify stimulus-locked responses.

Language dominance on fMRI was established by an experienced neuroradiologist based on fMRI activation maps and quantified using voxel-count laterality index (LI). A region of interest (ROI) covering BA was defined based on the activation on correlation maps using anatomical (3DT1-weighted) images as reference for the IFG. LI was calculated based on the left (L) and right (R) activation volume of the ROI, using the formula: (L−R)/(L + R) [24]. Subjects were defined as having atypical language representation (right- or co-dominant) if their values fell between −1 and 0.2 [25].

### 2.5. Functional Connectivity and Graph Theory

We reconstructed individual language networks using functional ROIs obtained from task-based fMRI maps by applying optimal percolation thresholding, as shown in other published work [15,16,26,27]. Briefly, active voxels that were contiguous and in the same anatomical regions were labeled as belonging to the same functional ROI (Tables S2–S6). Functional links were inferred by thresholding pair-wise correlations between pair of voxels with a penalization parameter set to optimize brain integration (all the clusters are connected) and sparsity (minimal wiring), a technique known as optimal percolation [26]. This approach is based on the observation of biological systems and demonstrated promising results in the identification of meaningful connections [15,27]. We quantified the change of hemispheric connections over time with a ‘Connectivity laterality Index’ (CI). We used weighted values from connectivity matrices to account for connection strength. CI was calculated with the formula: (WC_L_)/(WC_L_ + WC_R_) where WC_L_ = total weight of left hemispheric links, WC_R_ = total weight of right hemispheric links. The CI ranges from 0 (only right intra-hemispheric connections) to +1 (only left intra-hemispheric connections), with 0.5 representing perfectly balanced connections between the two hemispheres. The analysis was conducted in Matlab (R2017a, Massachusetts: The MathWorks Inc., Natick, MA, USA).

### 2.6. Statistical Analysis

Functional correlation maps were generated at minimum threshold of *r* > 0.5 (uncorrected *p* = *2* × 10^−11^, *t* > 4, FDR-adjusted *p* value < 0.001), and used for conjunction analysis. To reduce false positives, voxels with standard deviation of the acquired time-series exceeding 8% of the mean signal intensity were set to zero.

We assessed the trend of the intra-patient correlation of LI and CI values, as well as language performance scores at the four time points with a linear mixed model (LMM) or linear model, when no cluster effects were detected. Correlation between LI, CI, patient’s age and language performance scores was also evaluated with LMM or linear model. Statistical significance threshold was set at *p* < 0.05 for every analysis.

## 3. Results

### 3.1. Cohort Clinical Description

**Figure 1:**
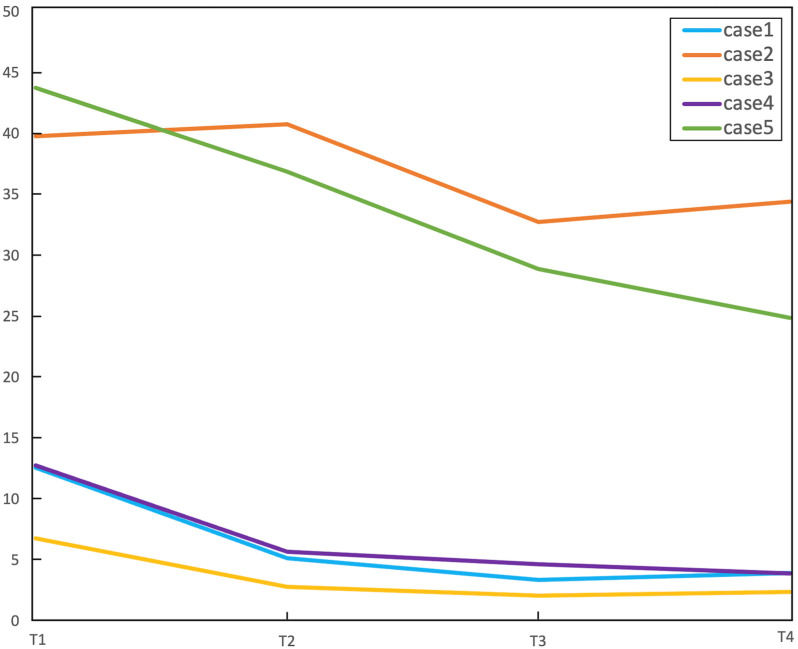
Tumor size over time for the five patients included in the study. Tumor size was measured as the product of the two greatest diameters on FLAIR images (cm^2^, y axis). Case 2 and 5 (described as possible ‘tumor-induced’ plasticity) presented larger tumor size at pre-op. T1 = baseline, T2 = post-op1 (4–8 months), T3 = post-op2 (10–14 months), T4 = post-op3 (16–23 months).

Five 100% right-handed patients (four males, mean age 47.6 years) with left-hemispheric LGG were included. Tumors involved the frontal lobe (3/5), insula (4/5) and temporal lobe (1/5). BA was involved by three/five tumors, Wernicke’s area (WA) by one/five tumors. Demographic data and tumor features are summarized in Table 1. Tumor size over time is reported in Figure 1. The extent of resection was 75% (case 1), 21% (case 2), 60% (case 3), 56% (case 4) and 43% (case 5). No radiologic progression was seen on MRI over the follow-up period. The five subjects’ ages were 31, 68, 50, 55 and 35 years, respectively, at the time of operation. When we included age in the linear mixed model, it was not significant (*p* = 0.81).

Intra-operative stimulation was obtained during the surgery for every case. Speech arrest at the location of the fMRI-identified BA was reported in four/five patients (case 1, 2, 3, 4). One case showed a lack of speech arrest at pre-op (case 5), despite extended stimulation to the area of the frontal operculum surrounding the tumor.

Language assessment with BNT, PVF and CF test was obtained for every timepoint in two/five patients. Two/five patients lacked one timepoint, while one/five patient lacked two timepoints, due to clinical condition and/or a lack of compliance (Table S1). The statistical analysis of the language test performance using LMM demonstrated no significant changes over time (BNT *p* = 0.48, PVF *p* = 0.62, CF *p* = 0.82). One patient (case 2) had z-scores < 1.5 standard deviations below the normative mean in every test and timepoint. (Table S1).

Patients received standard treatment after surgery including chemotherapy and radiation: four/five patients (case 1, 2, 3, 5) received radiotherapy between the pre-op and the post-op1 timepoint of the study (5400–5940 cGy, 180 cGy/fraction, 30/33 fractions), two/five patients (case 2, 5) also received chemotherapy between the post-op2 and post-op3 timepoints of the study (Temozolomide, 5/6 cycles). None of the patients received treatment before the first timepoint of the study. The treatment data is summarized in Table 2.

### 3.2. fMRI Activation Maps and Laterality Analysis

**Figure 2:**
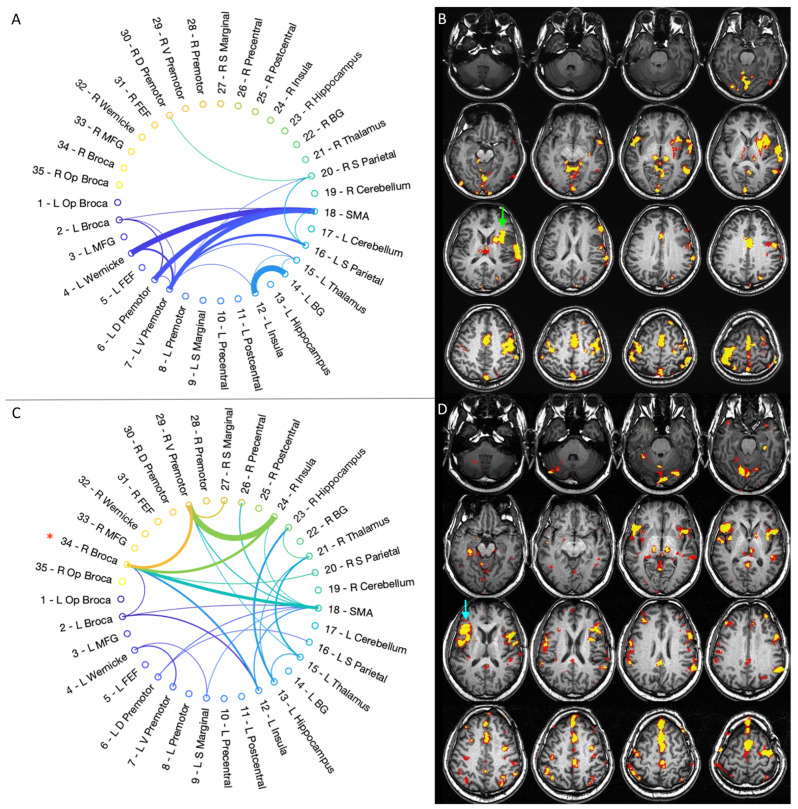
Representative case for type 1 language reorganization. The images on top show the connectivity diagram (**A**) and fMRI activation map obtained with letter task (**B**) at the first timepoint (pre-op). The patient was left-dominant on the preoperative fMRI, with strong activation of Broca’s area (green arrow in (**B**)). The connectivity diagram mirrors this finding, showing predominant left-hemispheric connections. The images at the bottom show the connectivity diagram (**C**) and fMRI activation map obtained with letter task (**D**) at the last timepoint (post-op3). The patient displayed right-dominance on fMRI, with strong activation of right Broca’s area homologous (light blue arrow in (**D**)). The connectivity diagram shows increased inter-hemispheric and right-side connections, including Broca’s area homologous (red asterisk in (**C**)). This type of reorganization was found in case 1, 3, 4.

Four MRI scans including language fMRI with phonemic fluency task were obtained for every patient, with the following intervals: pre-op, post-op1 (4–8 months), post-op2 (10–14 months) and post-op3 (16–23 months). A visual inspection of the fMRI maps demonstrated a predominant left activation in three/five patients at the pre-op timepoint (case 1, 3, 4). A progressive increase in right-sided activation over time was noted in all of these cases, including BA and WA homologues (Figure 2).

**Figure 3:**
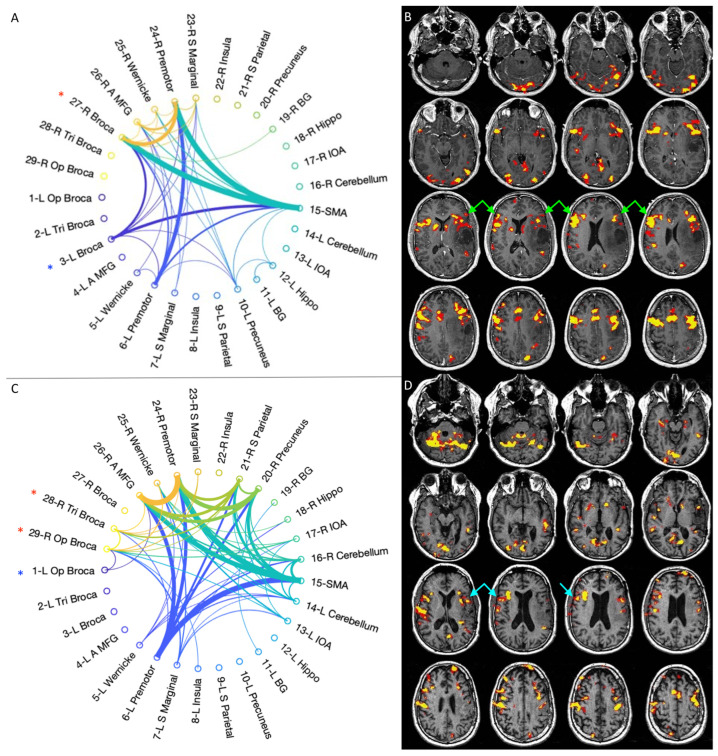
Representative case for type 2 language reorganization. The images on top show the connectivity diagram (**A**) and fMRI activation map obtained with letter task (**B**) at the first timepoint (pre-op). The patient was right-dominant on the preoperative fMRI, with right-greater-than-left activation of Broca’s area (green arrows in (**B**)). The connectivity diagram mirrors these findings, showing stronger connectivity in right Broca’s area homologous (red asterisk in (**A**)) than in left Broca’s area (blue asterisk in (**A**)). The images at the bottom show the connectivity diagram (**C**) and fMRI activation map obtained with letter task (**D**) at the last timepoint (post-op3). The patient remained right-dominant on fMRI (light blue arrows in (**D**)). The connectivity diagram shows increased inter-hemispheric and right-side connections, with persistent stronger connectivity in right Broca’s area homologous (red asterisks in (**C**)) than in left Broca’s area (blue asterisk in (**C**)). This type of reorganization was found in case 2, 5.

Conversely, two/five patients showed more-than-expected right-sided language-related activation at the pre-op timepoint, particularly involving BA homologous. The atypical activation was maintained up to the last post-operative timepoint (Figure 3). Details about active areas in every subject, individual connectograms and fMRI maps for every timepoint are provided as Supplemental Material.

**Figure 4:**
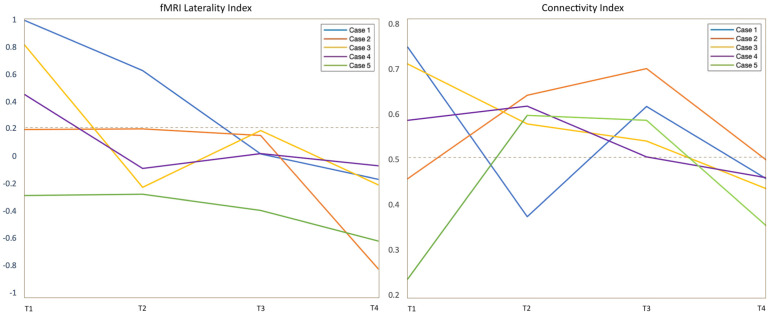
The left panel shows the fMRI laterality index for all cases across the four timepoints of the study (T1–T4). Values above 0.2 were considered left dominant. The right panel shows the connectivity laterality index for all cases across the four timepoints of the study (T1–T4). The closer the values are to 0.5, the more balanced is the connectivity between the two hemispheres. Values above 0.5 point to higher participation of the left hemisphere. LI obtained from tb-fMRI showed significant left-to-right trend in all patients across timepoints.

fMRI LI demonstrated decreased left-dominance over time in three/five patients (Figure 4): case 1 changed from LI = 1 (pre-op) to LI = −0.17 (post-op3), case 3 changed from LI = 0.81 (pre-op) to LI = −0.20 (post-op3) and case 4 changed from LI = 0.45 (pre-op) to LI = −0.07 (post-op3). In two/five patients, the analysis demonstrated co-/right-dominance across scans. These baseline atypical-dominant patients still showed a trend of increased right-activation over time, demonstrated by a corresponding decrease in LI: case 2 changed from LI = 0.19 (pre-op) to LI = −0.82 (post-op3); case 5 changed from LI = −0.28 (pre-op) to LI = −0.61 (post-op 3). The linear mixed model used to compare LI values in every patient confirmed a significant decreasing trend (*p* < 0.001). No significant correlation was found between LI values and language performance (BNT *p* = 0.19, PVF *p* = 0.64, CF *p* = 0.21).

### 3.3. Graph-Theory Analysis

**Figure 5:**
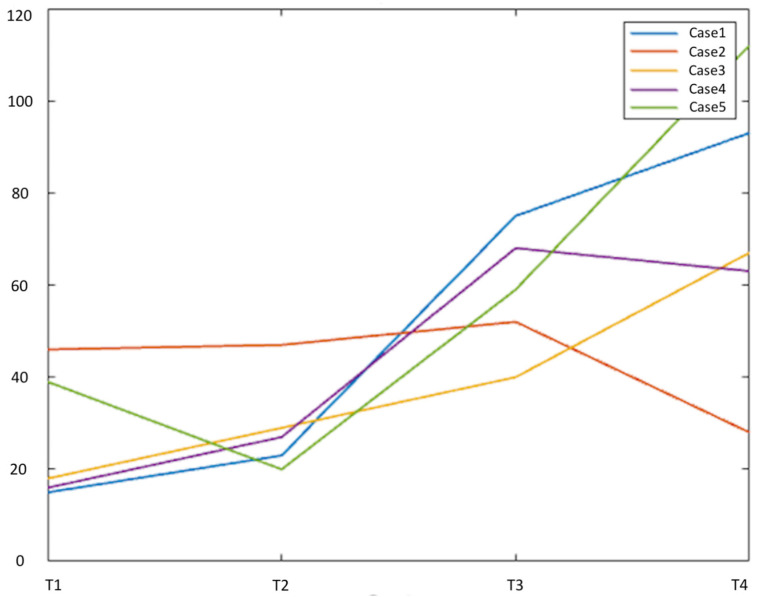
Graphical representation of the total number of intra- and inter-hemispheric connections between active fMRI clusters across the four timepoints of our study. Each case is represented as a colored line (legend in the figure). The number of links is depicted in the y-axis, while the timepoints are shown on the x-axis of the graph. All cases demonstrated an increased number of links from the first to the last timepoint, with the exception of case 2 (orange line).

Connectivity diagrams obtained through an optimal percolation technique showed an increased total number of intra- and inter-hemispheric links over time in four/five patients, especially when comparing the first to the last timepoint (Figure 5).

This observation was more evident for patients starting as strongly left-dominant and developing laterality shift on fMRI (case 1, 3, 4). Case 2 was the only one displaying a slightly decreased total number of connections from the first to the last timepoint. Besides increased whole-brain connectivity, the diagrams showed enhanced right-sided connections intra- and inter-hemispheres across scans, including language-related areas: BA (five/five), WA (three/five), premotor cortex (two/five), middle frontal gyrus (five/five), insula (three/five). Individual connectograms for every timepoint are available as Supplemental Material. A quantification of left and right-sided connections through the CI is provided in Figure 4.

The CI evaluation partly mirrored the results of the fMRI LI. Three/five patients showed left-greater-than-right connections at the pre-op timepoint, with a right-shift at the last timepoint: case 1 changed from 0.75 to 0.46 on the last follow-up; case 3 shifted from 0.71 to 0.44; and case 4 changed from 0.59 to 0.46 (example in Figure 2)**.** Two/five cases showed more-than-expected right-sided connections at the pre-op timepoint: case 2 presented CI of 0.46 at the first timepoint, changing to 0.50 on post-op 3; case 5 demonstrated CI of 0.24 initially and shifted to 0.35 on post-op 3 (0.5 represents perfectly balanced connections between the hemispheres) (Figure 3). However, the statistical analysis of CI values did not confirm a significant decreasing trend (*p* = 0.27). No significant correlation was found between CI values and language performance (BNT *p* = 0.29, PVF *p* = 0.97, CF *p* = 0.11).

The fMRI and connectivity results for every patient and timepoint are reported in the Supplementary Material (Figures S1–S20).

## 4. Discussion

We demonstrated the gradually increased involvement of the right hemisphere in language function over time in patients with left-hemispheric LGG. We found two patterns of plasticity: Type 1 in which three/five patients showed strong left dominance on the pre-operative scan, followed by a shift to co-dominance after surgery; Type 2 in which two/five patients showed initial atypical dominance at the pre-operative timepoint, which persisted after surgery.

Language translocation over time in LGG patients may reflect different factors. In type 1 changes, treatments including surgery seem pivotal to develop the reorganization, since strong left-dominance was noted at the pre-op timepoint. Previous studies demonstrated that resection of brain tumors involving eloquent language areas in the dominant hemisphere may lead to reorganization, with possible effects on surgical outcome [28,29,30,31]. Chemotherapy may lead to structural and functional changes in the brain, as a correlate of cognitive deficits [32]. After recovery, these modifications may persist, supporting the idea that brain plasticity may at least partially compensate for the deleterious effect of chemotherapy [32]. Pre-operative fMRI is now considered state-of-the-art; however, post-operative fMRI is rarely performed. In our study, plastic changes were only visible through longitudinal evaluation in three/five patients. Such changes were somehow progressive, but best evidenced by comparing the pre-op scan with the post-op3 timepoint, more than one year after surgery. If confirmed in larger cohorts, our results would support the use of longitudinal fMRI after surgery to monitor the development of reorganization and tailor therapeutic interventions.

Type 2 functional changes may point to tumor-induced rather than surgery-induced plasticity. The existence of pre-surgical reorganization is confirmed by previous studies, including inter- and intra-hemispheric changes [6,33,34,35,36]. Rosenberg et al. reported the inter-hemispheric translocation of BA in a patient with LGG during the two years before surgery [37]. Our results appear to be in line with this evidence. In type 2 cases, the left-to-right shift of activation was less evident than in type 1. This may depend on different time-windows for surgery- vs tumor-induced functional changes, with the former being faster than the latter. Interestingly, patients with type 2 plasticity showed larger tumors than type 1 (Figure 1). Tumor pathology being equal, larger tumors may indicate longer disease duration, promoting functional changes before our first scan and baseline atypical dominance. Intra-operative stimulation results support our connectivity findings in type 2 patients: the subject with right-dominance for language at the pre-op timepoint (case 5) was the only one showing a lack of speech arrest despite extensive stimulation of BA, providing surgical confirmation of the fMRI findings.

Other considerations emerge from our analyses. The comparison of fMRI LI and CI provided interesting insights (Figure 4). Our results demonstrate that LGG and surgical changes induce a modification of language dominance characterized by the left-to-right shift of BA (visible on the fMRI LI). Additionally, the right hemisphere displays increased connectivity between language areas homologues (visible on CI). While the fMRI LI shows more local changes, the CI offers a different perspective on functional reorganization at the global level. The fMRI LI showed a significant consistent trend of right-shift across subjects. On the other hand, the CI did not show statistically significant trend. This was probably due to the non-uniform progression of functional changes from the first to the last timepoint, characterized by initial fluctuations after surgery. Such results highlight the importance of longitudinal fMRI to capture long-term vs transient changes in connectivity, which may depend on the immediate impact of surgical resection (‘stunned’ brain theory [38]).

Connectivity diagrams showed increased functional connections over time in four/five patients, including right-sided language areas-homologues (Figure 5). Increased connectivity and inter-hemispheric communication may represent a correlate of callosal disinhibition, one of the hypothesized mechanisms of contralateral reorganization [39]. The only patient with no increase in whole-brain connectivity over time was case 2, who performed in the impaired range on all language tests, raising the possibility of a relationship between whole-brain connectivity and language performance. Conversely, none of the patients showed significant changes in language test performance during the follow up period. Since the tumors did not show definite radiological progression, our data may support the idea that inter-hemispheric language translocation is not associated ‘per se’ with a clinical benefit, as suggested in previous studies [28]. Increased inter-hemispheric connectivity may constitute a permissive environment to develop plastic phenomena [40]. However, there might be other requirements besides the general increment in connectivity to produce a net clinical benefit. Another interesting hypothesis would be that the increased whole-brain connectivity, as well as the left-to-right shift, may contribute to preserve language function against the (potential) negative effects from the infiltrating glioma, surgery and chemo-radiation treatment (CRT). CRT may cause inflammation and demyelination and decrease neurogenesis, as well as promote hormonal changes [32], with effects on brain connectivity [41,42]. Plasticity may come into play to, at least partially, compensate for the cognitive side effects of these treatments, preventing further performance decline [32].

Our work has some limitations. First, the number of participating patients is small, decreasing the generalizability of our results. The limited number of subjects was due to strict inclusion criteria and the challenge of performing a multi-step longitudinal fMRI study. Particularly, the population of the study was limited to newly diagnosed right-handed LGG involving left peri-sylvian eloquent areas, with four timepoints of follow-up over a period of 18–24 months and no tumor progression in between scans. These strict requirements allowed us to evaluate language reorganization in a controlled population, excluding artifacts and confounding factors, at the cost of limiting the number of recruited subjects. As a consequence, the results of our study are intended as descriptive at the current stage, and need to be confirmed in larger populations. Clinical data is also limited, with some missing datapoints. Finally, additional studies would be required to assess the distinct contribution of radiotherapy (four/five patients) and chemotherapy (two/five patients) to language reorganization. The current study focused on the functional reorganization of language. However, language white matter pathways may also undergo plastic modifications in the setting of brain tumors, which could facilitate the compensation of deficits after damage of eloquent cortices [43]. Future studies should explore the longitudinal reorganization of white matter pathways in brain tumor patients.

## 5. Conclusions

Our study demonstrates the longitudinal development of language plasticity in patients with LGG. Two patterns of language reorganization were identified: type 1 changes may be related to treatment, including surgery; type 2 may be tumor-induced, since atypical language organization was already present at the pre-op timepoint. Increased inter-hemispheric connectivity may represent the initial step in the development of plastic phenomena. This change could be partially compensatory; however, other factors might be required to improve language performance.

## Figures and Tables

**Table 1:** Patients’ demographics and tumor features.

Case	Hand	Sex	T1 (D)	T2 (M)	T3 (M)	T4 (M)	Tumor Diagnosis	Location	Eloquent Areas	IOS
1	R	F	60	8	6	9	astro WHO2	3		1
2	R	M	1	4	6	7	astro WHO2	2,3,4	2	1
3	R	M	30	7	4	6	oligo WHO2	1	1	1
4	R	M	1	4	7	6	oligo WHO2	1,3	1	1
5	R	M	2	4	6	6	astro WHO2	1,3	1	2

T1 = pre-op timepoint, days before surgery; T2 = post-op2 timepoints, months after T1; T3 = post-op3 timepoints, months after T2; T4 = post-op4 timepoints, months after T3; astro = astrocytoma; oligo = oligodendroglioma; WHO = world health organization classification; Location: 1 = frontal, 2 = temporal, 3 = insular, 4 = basal ganglia; eloquent areas: 1 = Broca’s, 2 = Wernicke’s; IOS = intra-operative stimulation, 1 = speech arrest, 2 = no speech arrest.

**Table 2:** Treatment received by our patients during the follow-up period.

	RT Follow-Up Interval	RT Fractions	RT Total Adjuvant Dose/Energy	CT Follow-Up Interval	CT Regime
**Case 1**					
	T1–T2	30	Total 5400 cGy, 180 cGy/fraction, 6 MV	/	/
**Case 2**					
	T1–T2	33	Total 5940 cGy, 180 cGy/fraction, 6 MV	T2–T3	Temozolomide, 5 cycles
**Case 3**					
	T1–T2	30	Total 5400 cGy, 180 cGy/fraction, 6 MV	/	/
**Case 4**					
	/	/	/	/	/
**Case 5**					
	T1–T2	33	Total 5940 cGy, 180 cGy/fraction, 6 MV	T3–T4	Temozolomide, 6 cycles

CT = chemotherapy; RT = radiotherapy; T1 = pre-op timepoint; T2 = post-op 1 timepoint; T3 = post-op 2 timepoint; T4 = post-op3 timepoint.

## Data Availability

Patients data is available upon request to the corresponding author and approval from the local ethics committee.

## Supplementary Materials

The following supporting information can be downloaded at: https://www.mdpi.com/article/10.3390/cancers15030836/s1, Table S1: Clinical evaluation of speech ability through neurocognitive tests; Table S2: Active clusters for patient 1 at different timepoints (threshold criteria *r* > 0.5; uncorrected *p* = 2 × 10^−11^); Table S3: Active clusters for patient 2 at different timepoints (threshold criteria r > 0.5; uncorrected *p* = 2 × 10^−11^); Table S4: Active clusters for patient 3 at different timepoints (threshold criteria r > 0.5; uncorrected *p* = 2 × 10^−11^); Table S5: Active clusters for patient 4 at different timepoints (threshold criteria r > 0.5; uncorrected *p* = 2 × 10^−11^); Table S6: Active clusters for patient 5 at different timepoints (threshold criteria r > 0.5; uncorrected *p* = 2 × 10^−11^); Figure S1: Connectivity diagram (left) and fMRI activation map for letter task (right) acquired before surgery at the first timepoint (pre-op) for case 1; Figure S2: Connectivity diagram (left) and fMRI activation map for letter task (right) acquired after surgery at the second timepoint (post-op1) for case 1; Figure S3: Connectivity diagram (left) and fMRI activation map for letter task (right) acquired after surgery at the third timepoint (post-op2) for case 1; Figure S4: Connectivity diagram (left) and fMRI activation map for letter task (right) acquired after surgery at the fourth timepoint (post-op3) for case 1; Figure S5: Connectivity diagram (left) and fMRI activation map for letter task (right) acquired before surgery at the first timepoint (pre-op) for case 2; Figure S6: Connectivity diagram (left) and fMRI activation map for letter task (right) acquired after surgery at the second timepoint (post-op1) for case 2; Figure S7: Connectivity diagram (left) and fMRI activation map for letter task (right) acquired after surgery at the third timepoint (post-op2) for case 2; Figure S8: Connectivity diagram (left) and fMRI activation map for letter task (right) acquired after surgery at the fourth timepoint (post-op3) for case 2; Figure S9: Connectivity diagram (left) and fMRI activation map for letter task (right) acquired before surgery at the first timepoint (pre-op) for case 3; Figure S10: Connectivity diagram (left) and fMRI activation map for letter task (right) acquired after surgery at the second timepoint (post-op1) for case 3; Figure S11: Connectivity diagram (left) and fMRI activation map for letter task (right) acquired after surgery at the third timepoint (post-op2) for case 3; Figure S12: Connectivity diagram (left) and fMRI activation map for letter task (right) acquired after surgery at the fourth timepoint (post-op3) for case 3; Figure S13: Connectivity diagram (left) and fMRI activation map for letter task (right) acquired before surgery at the first timepoint (pre-op) for case 4; Figure S14: Connectivity diagram (left) and fMRI activation map for letter task (right) acquired after surgery at the second timepoint (post-op1) for case 4; Figure S15: Connectivity diagram (left) and fMRI activation map for letter task (right) acquired after surgery at the third timepoint (post-op2) for case 4; Figure S16: Connectivity diagram (left) and fMRI activation map for letter task (right) acquired after surgery at the fourth timepoint (post-op3) for case 4; Figure S17: Connectivity diagram (left) and fMRI activation map for letter task (right) acquired before surgery at the first timepoint (pre-op) for case 5; Figure S18: Connectivity diagram (left) and fMRI activation map for letter task (right) acquired after surgery at the second timepoint (post-op1) for case 5; Figure S19: Connectivity diagram (left) and fMRI activation map for letter task (right) acquired after surgery at the third timepoint (post-op2) for case 5; Figure S20: Connectivity diagram (left) and fMRI activation map for letter task (right) acquired after surgery at the fourth timepoint (post-op3) for case 5.
