# Supplementary material for: Longitudinal Evaluation of Brain Plasticity in Low-Grade Gliomas: fMRI and Graph-Theory Provide Insights on Language Reorganization
Source: Cancers (Basel). 2023 Jan 29;15(3):836. doi: 10.3390/cancers15030836 (PMC9913404; doi:10.3390/cancers15030836)
Supplement: Supplementary file 1 [file cancers-15-00836-s001.zip › cancers-2105198-supplementary.pdf]

Figure S1. Case1 pre-op.

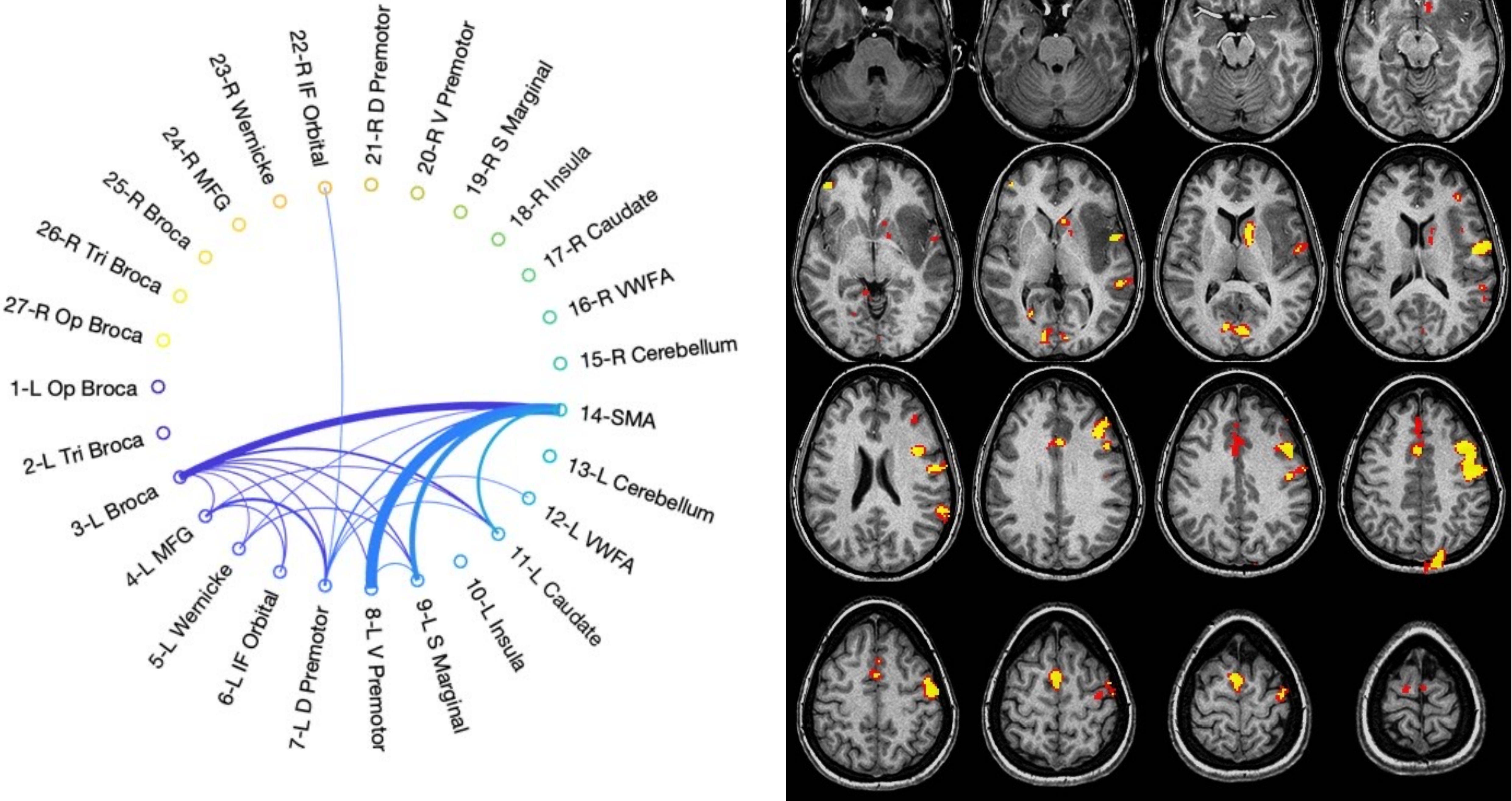

Figure S2. Case1 post-op1.

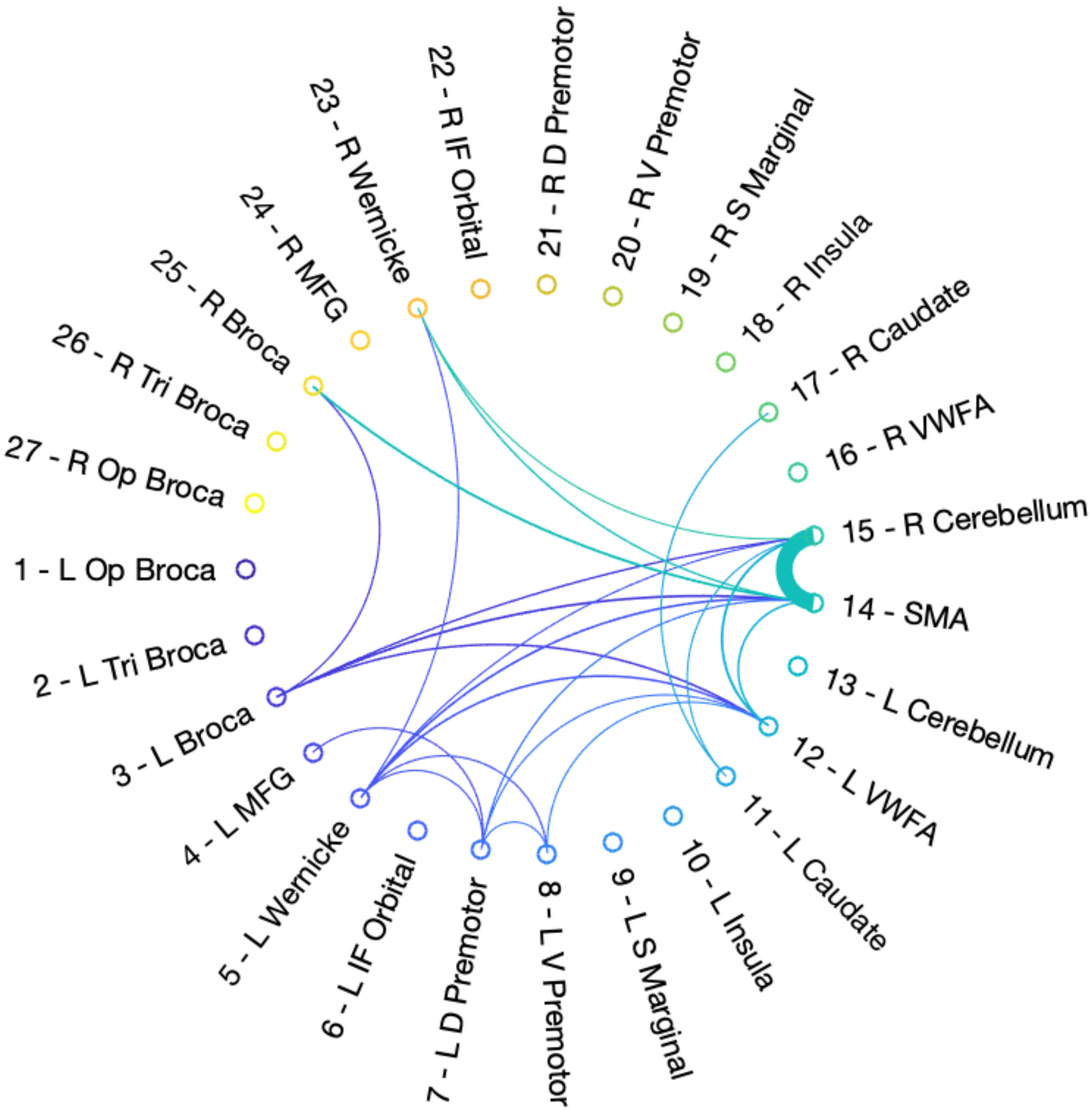

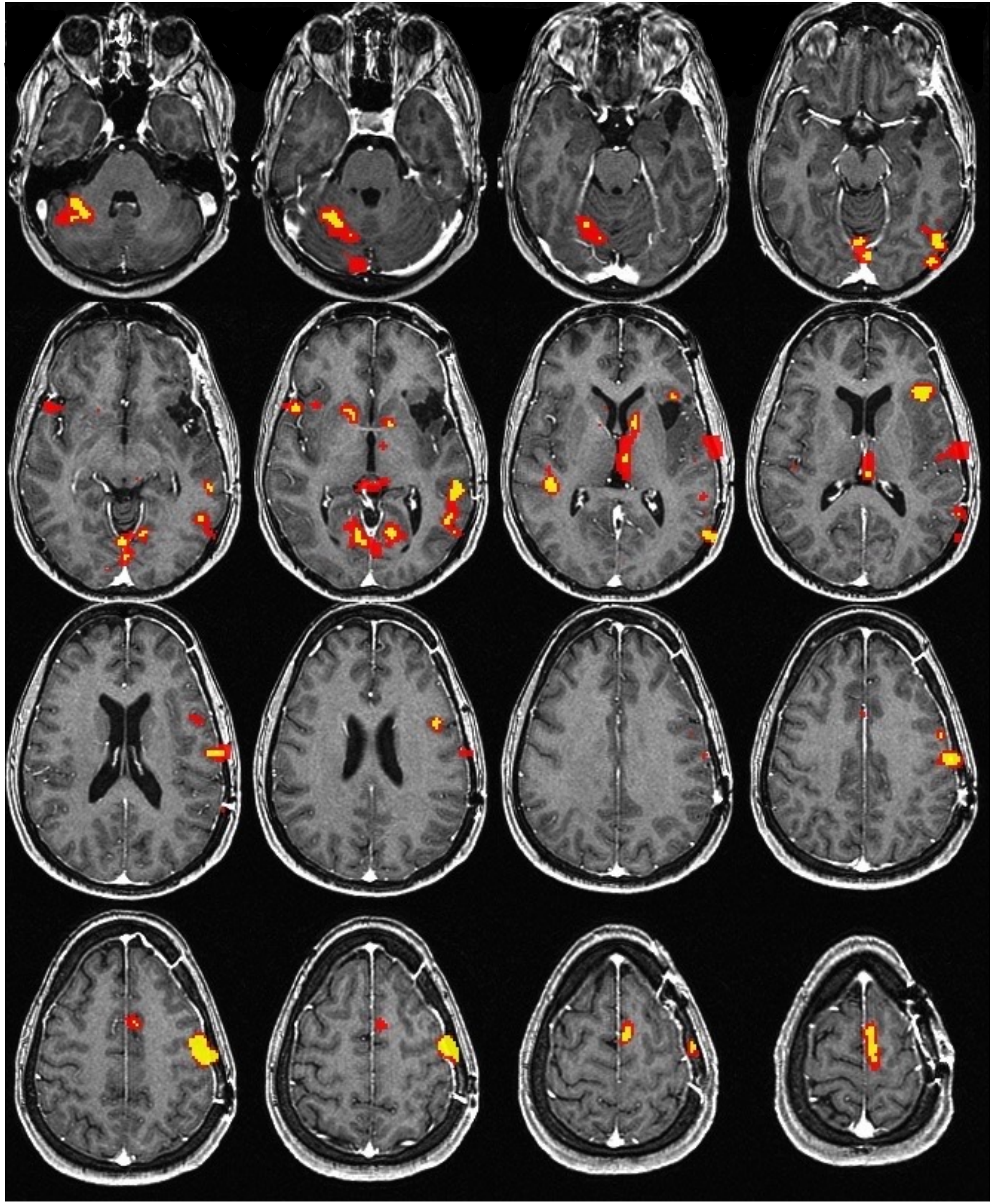

Figure S3. Case1 post-op2.

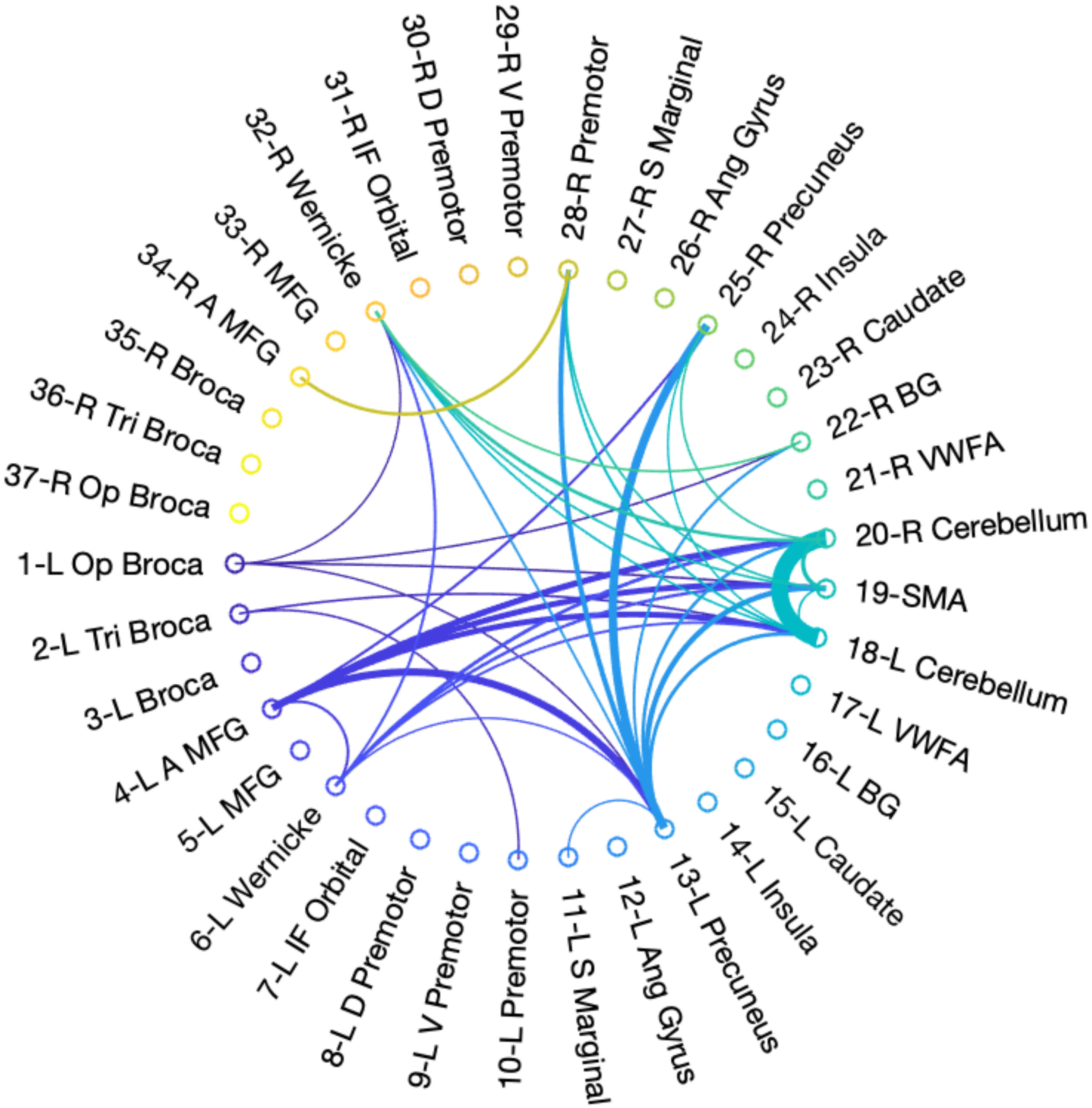

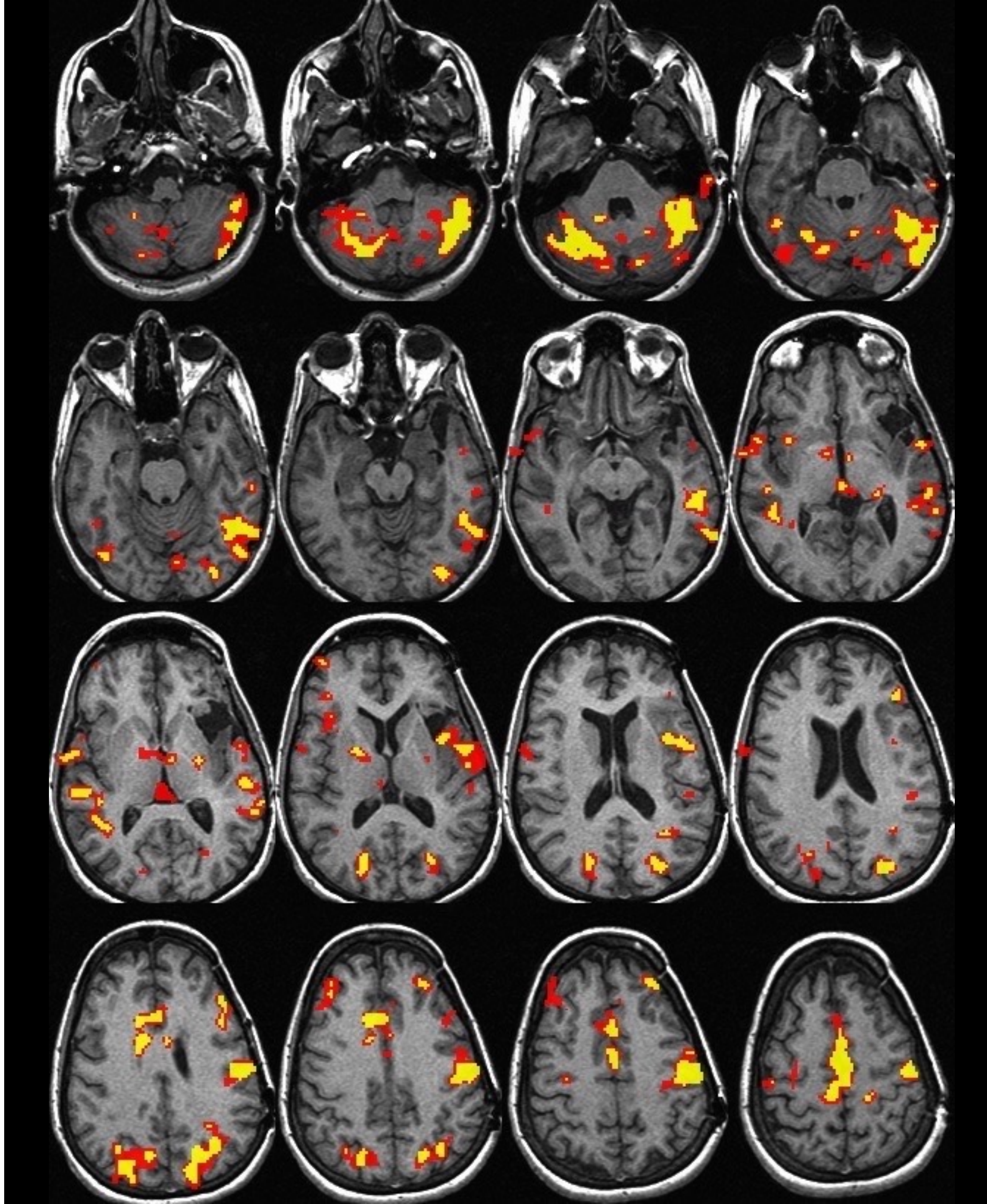

Figure S4. Case1 post-op3.

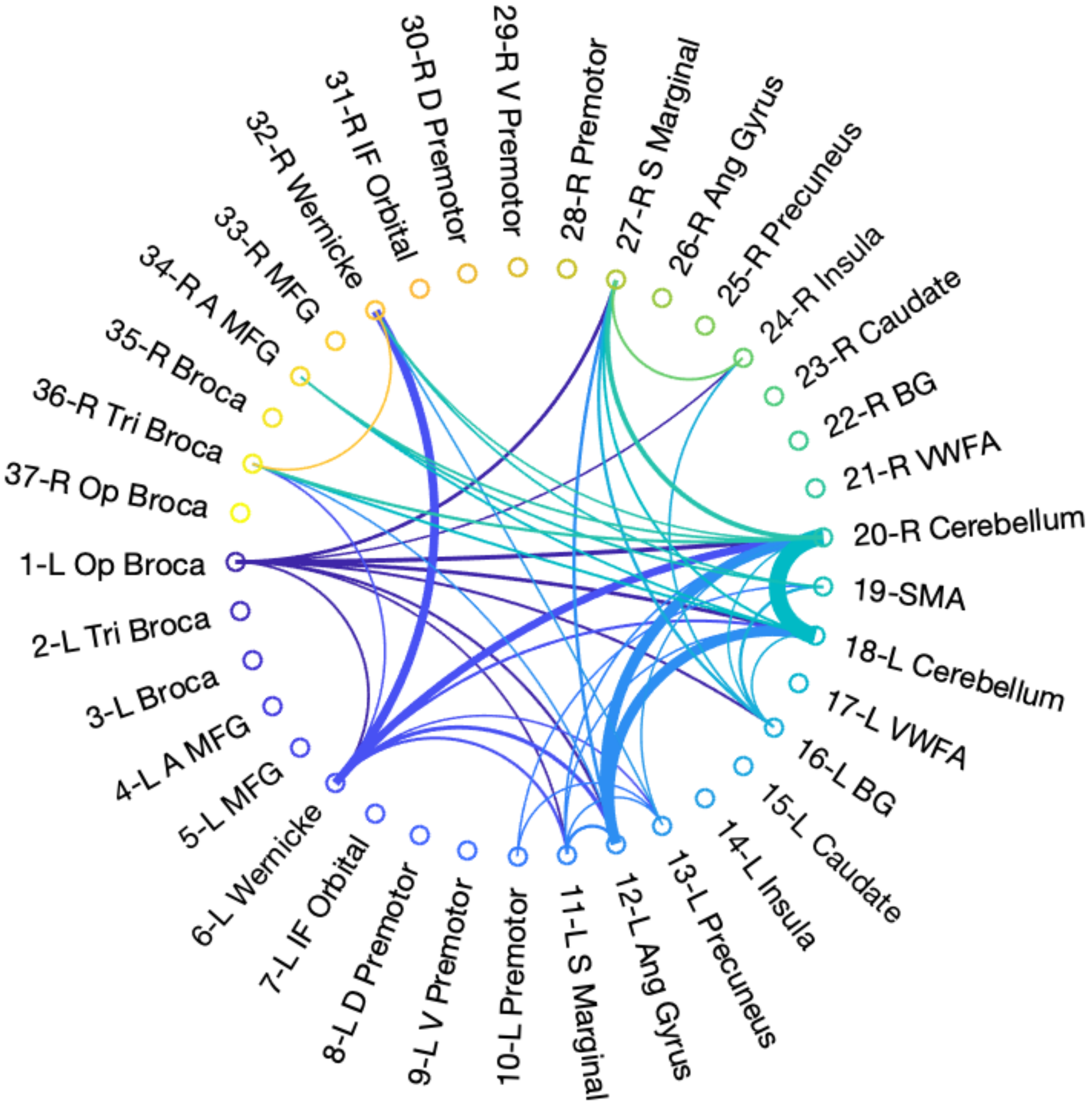

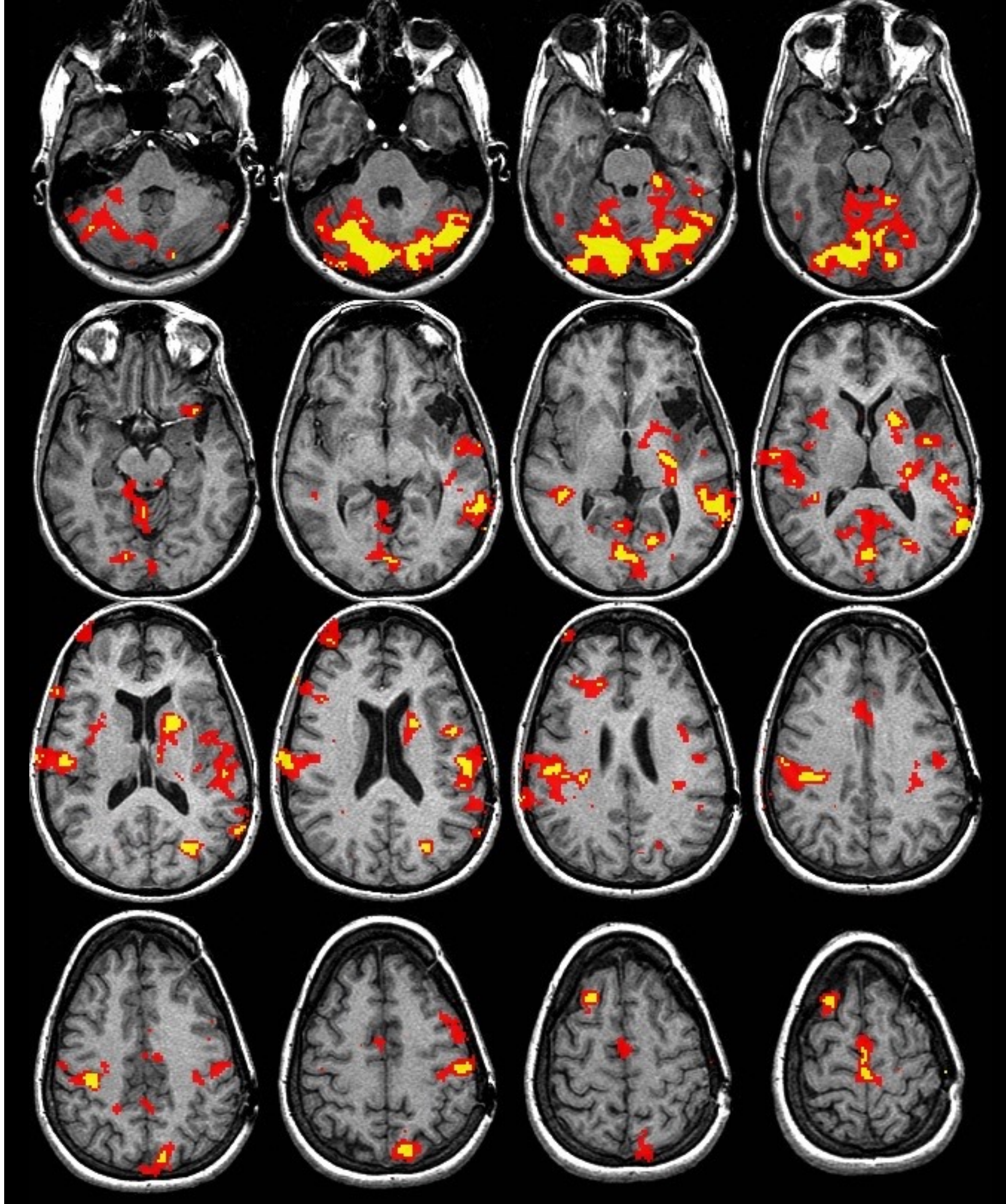

Figure S5. Case2 pre-op.

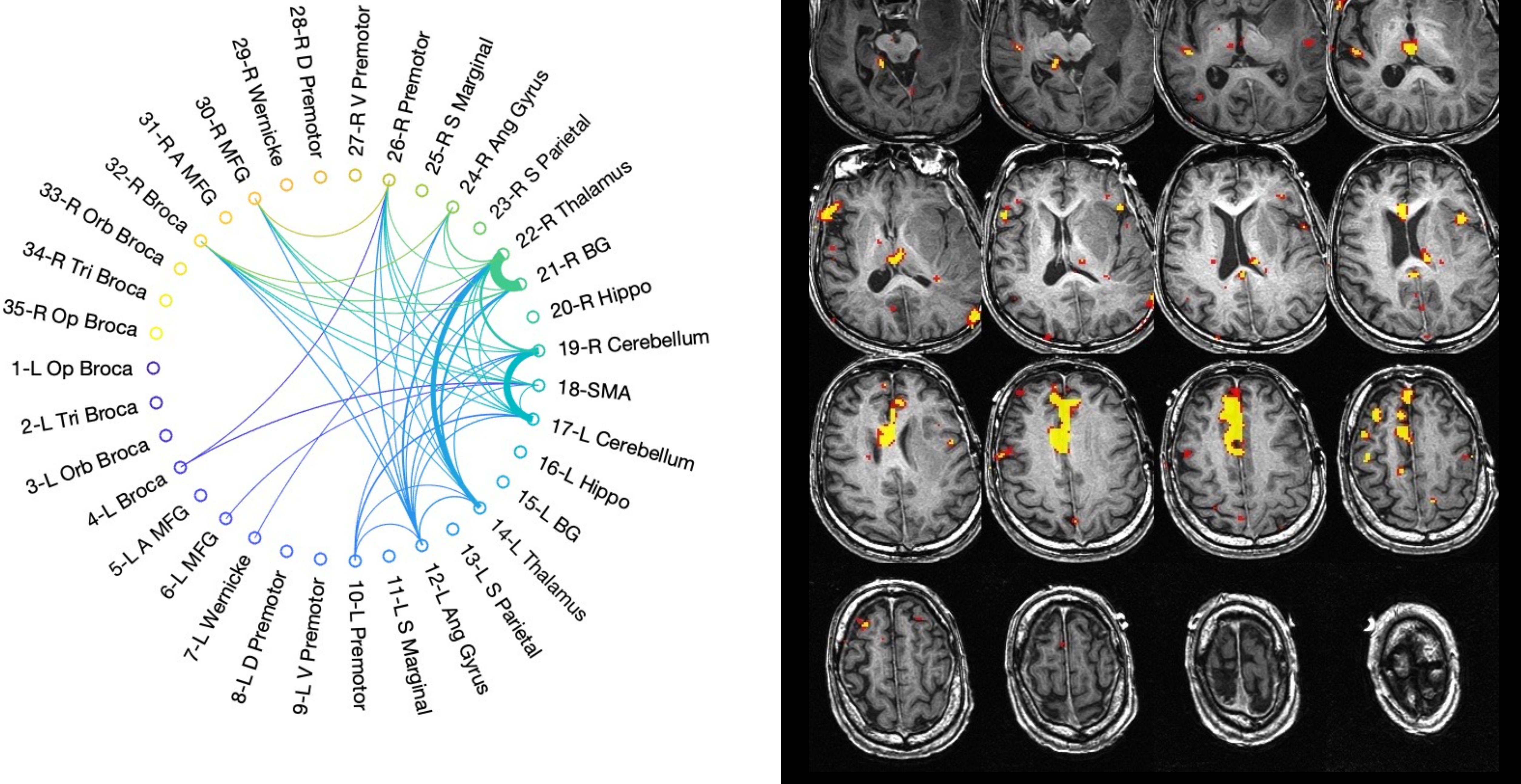

Figure S6. Case2 post-op1.

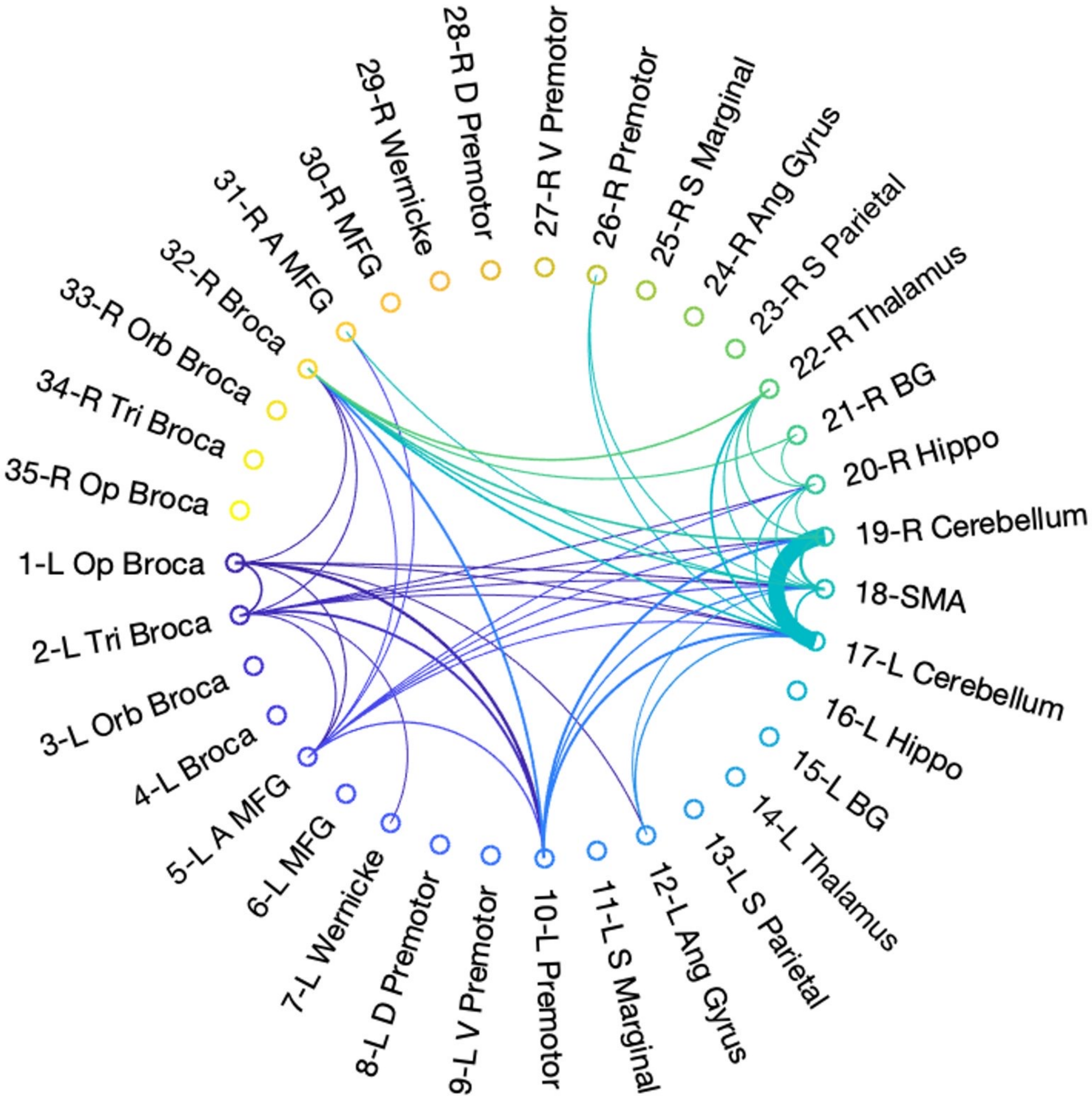

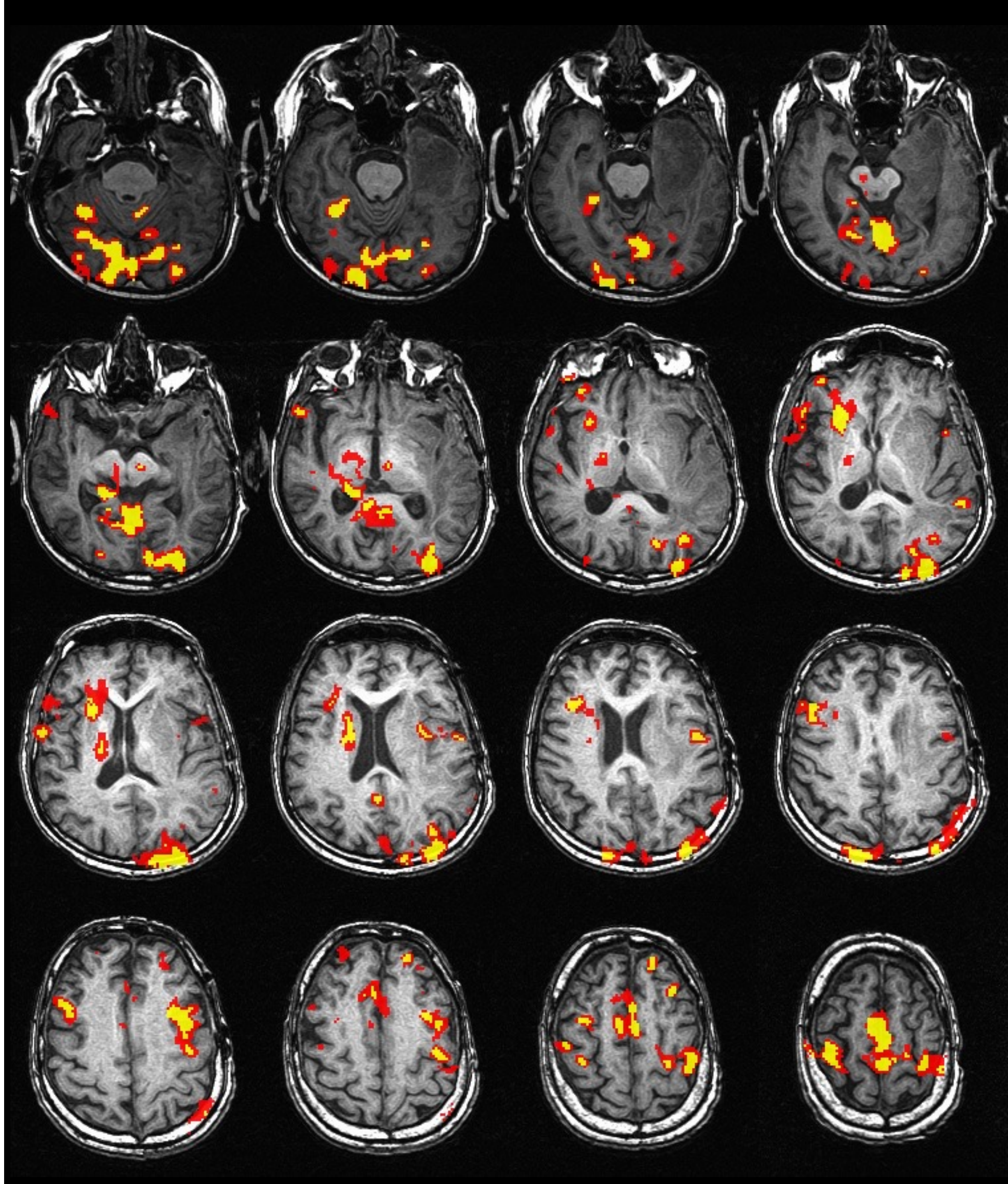

Figure S7. Case2 post-op2.

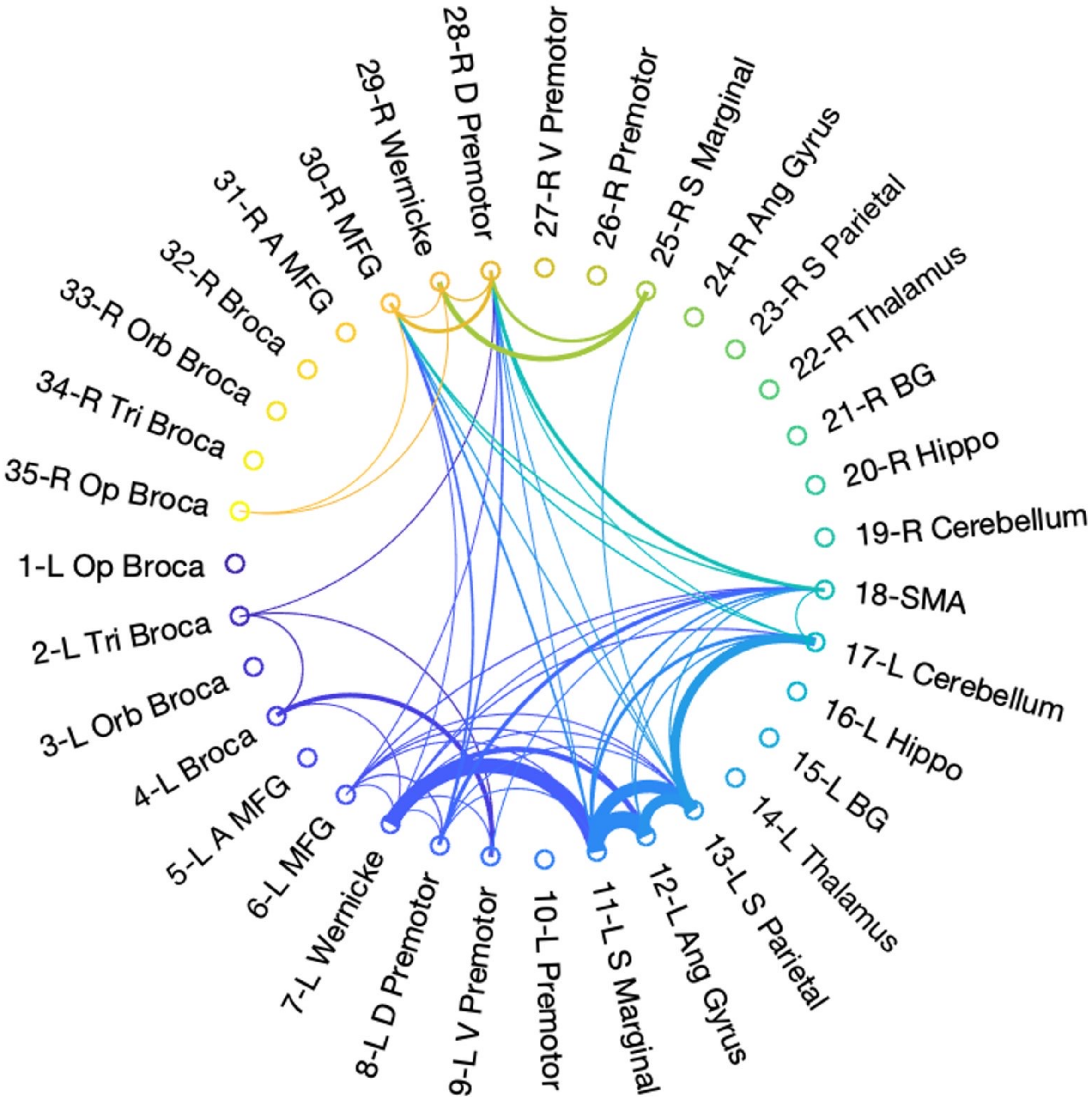

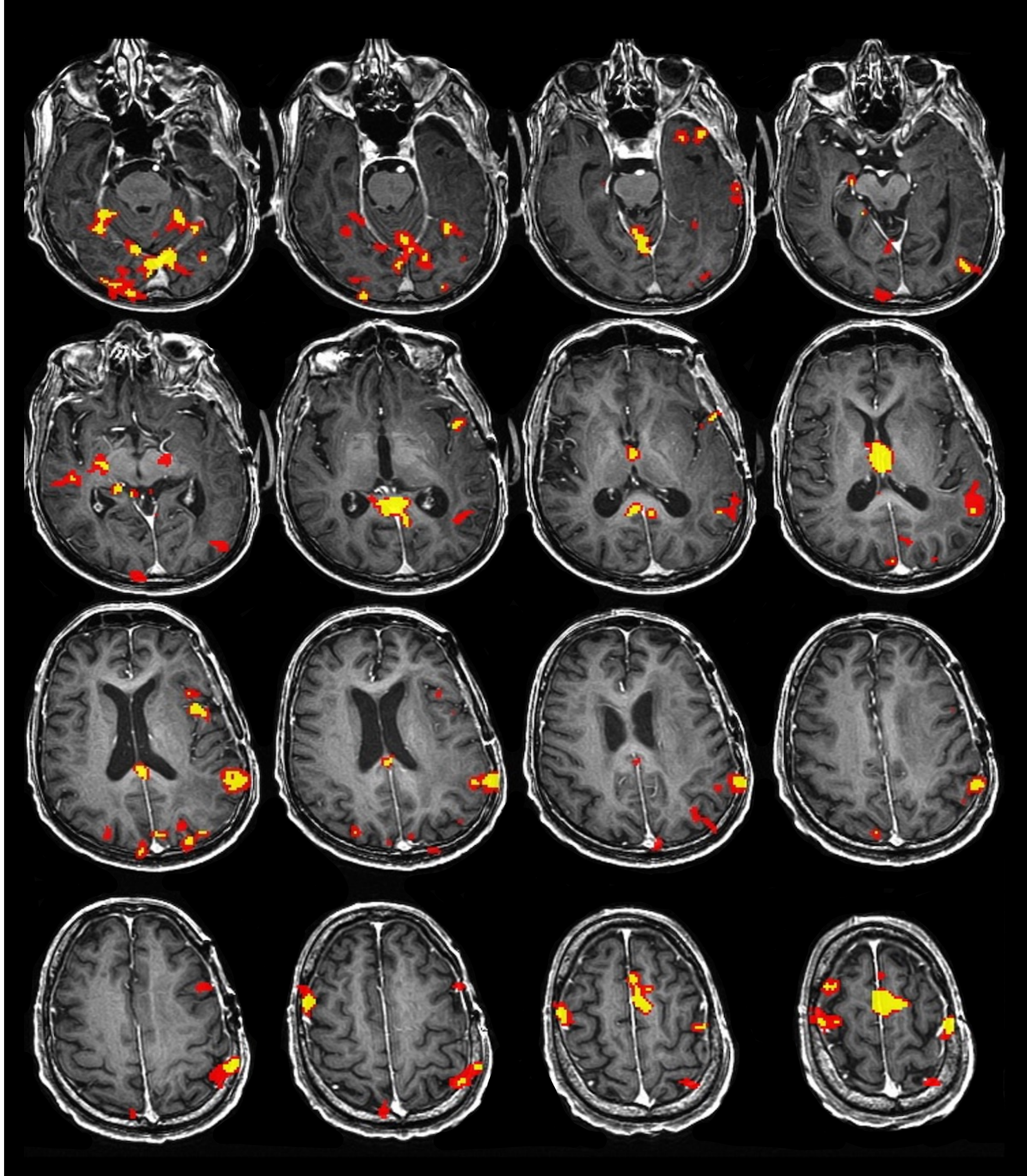

Figure S8. Case2 post-op3.

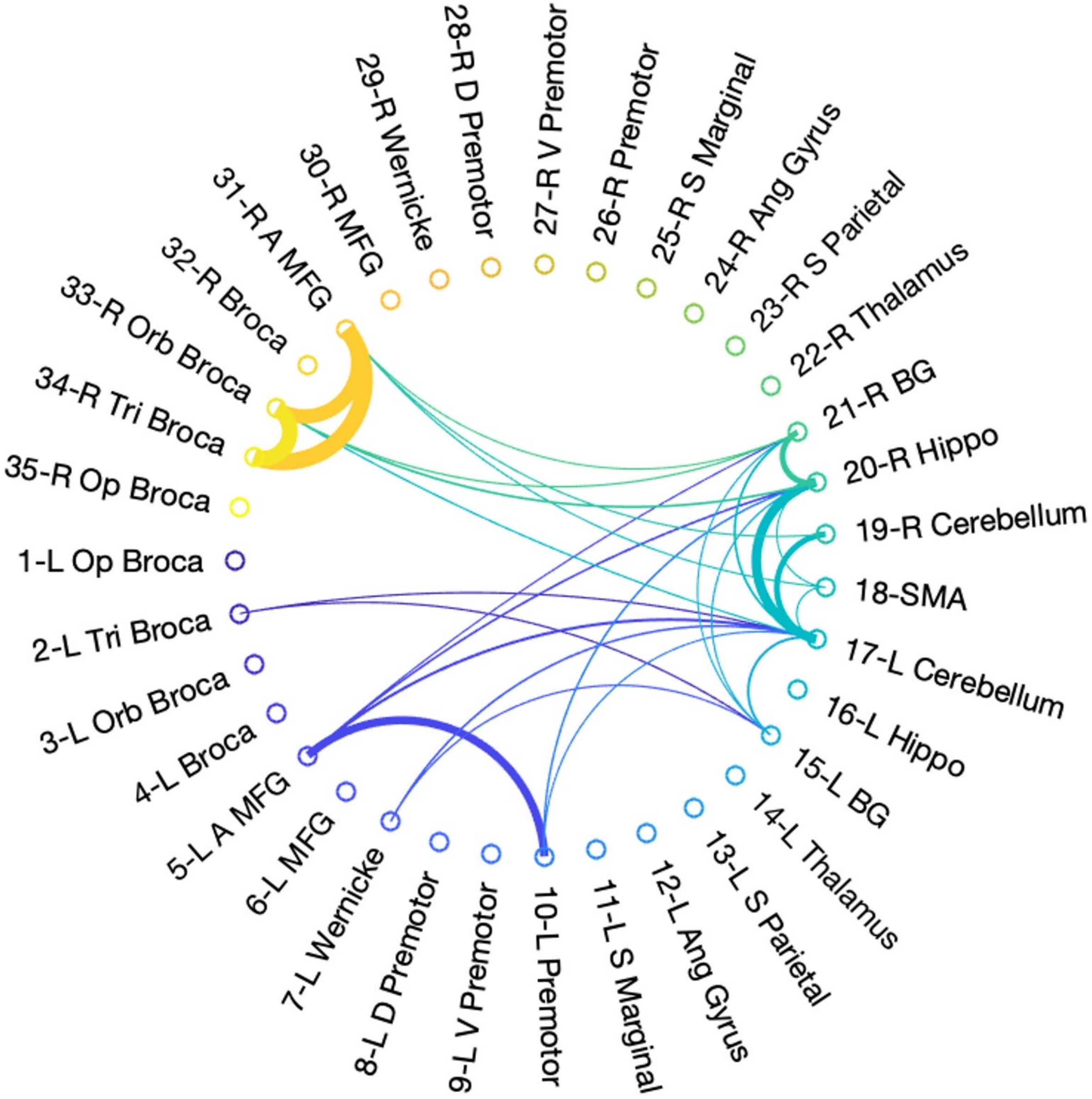

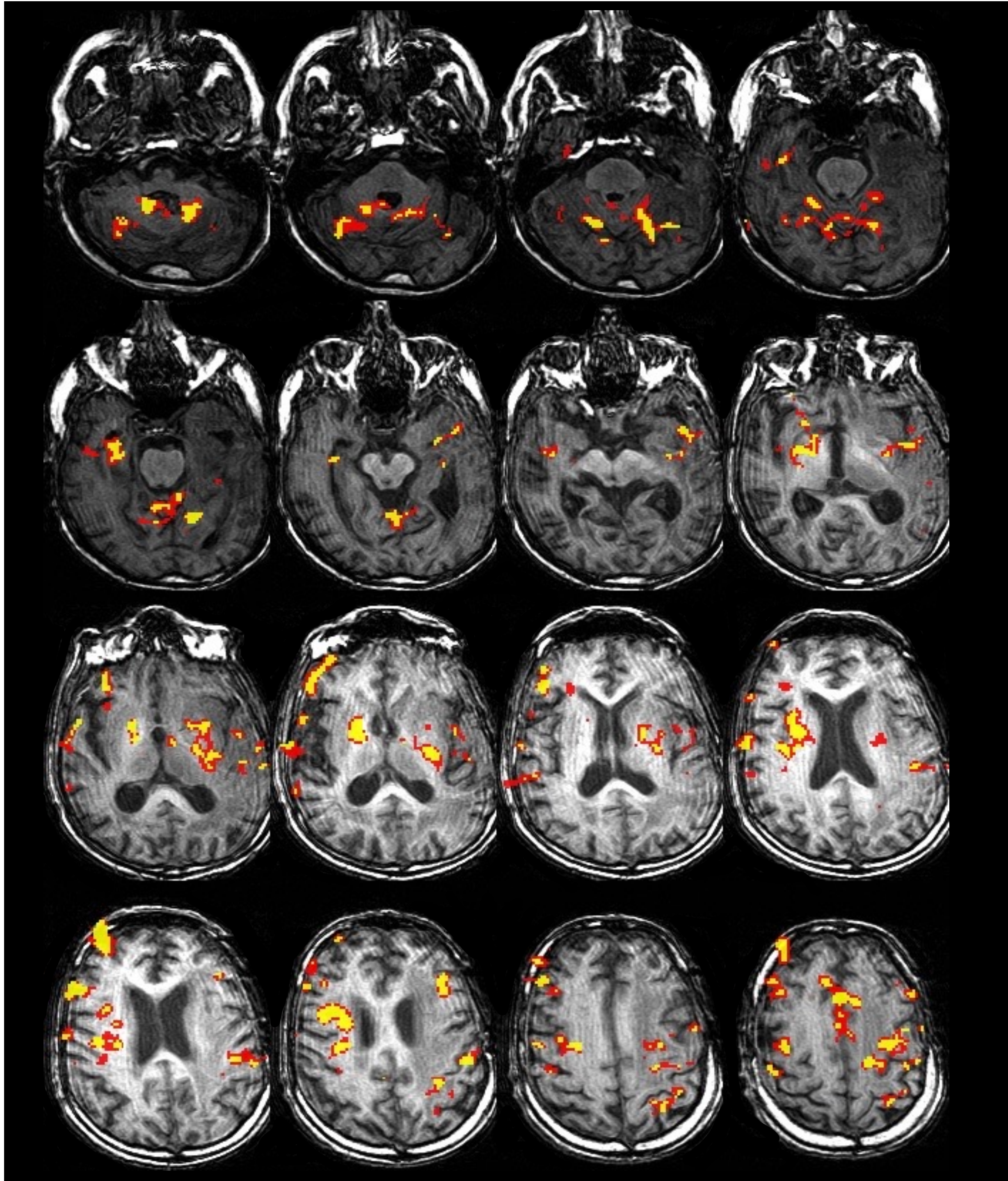

Figure S9. Case3 pre-op.

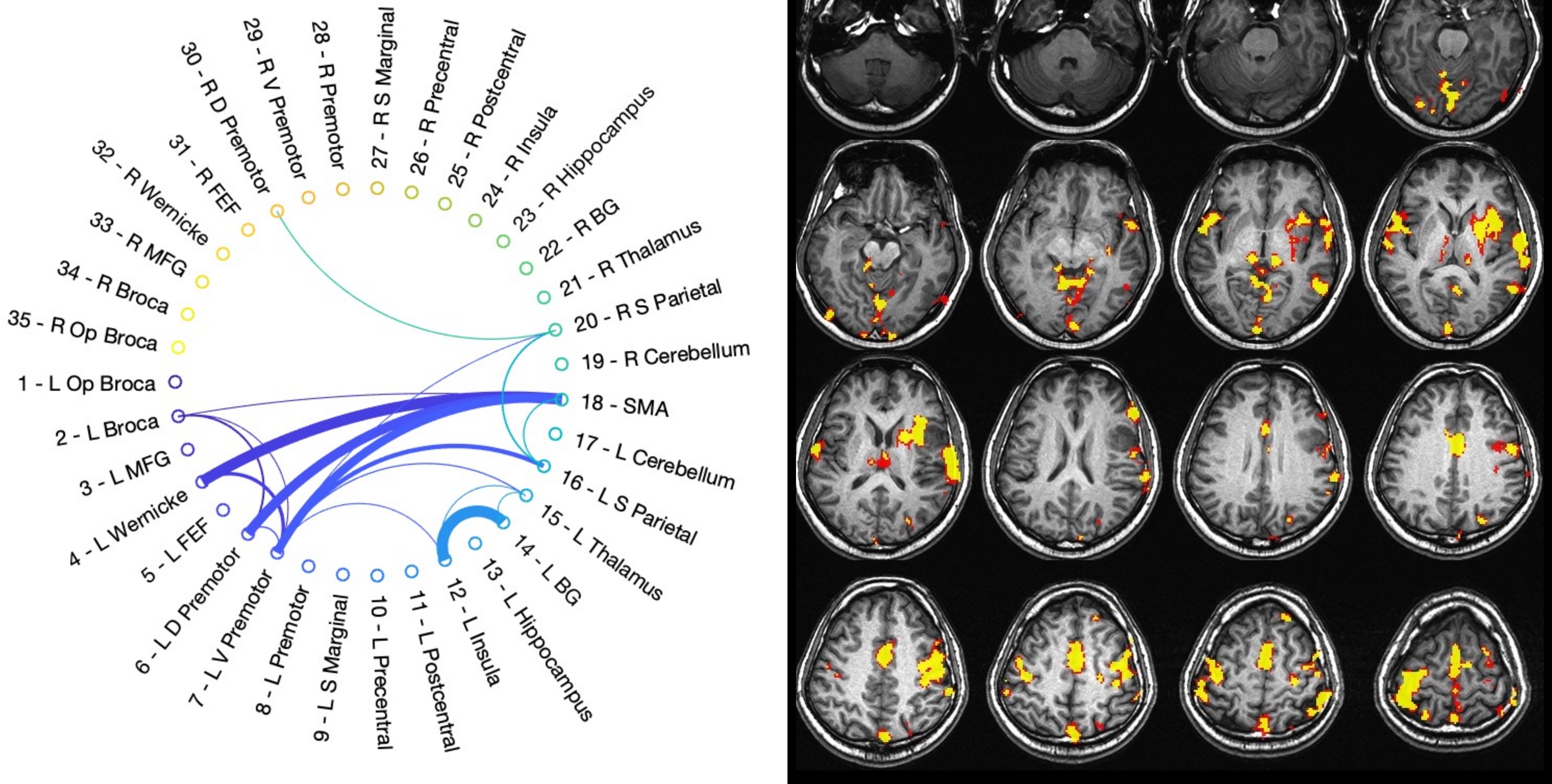

Figure S10. Case3 post-op1.

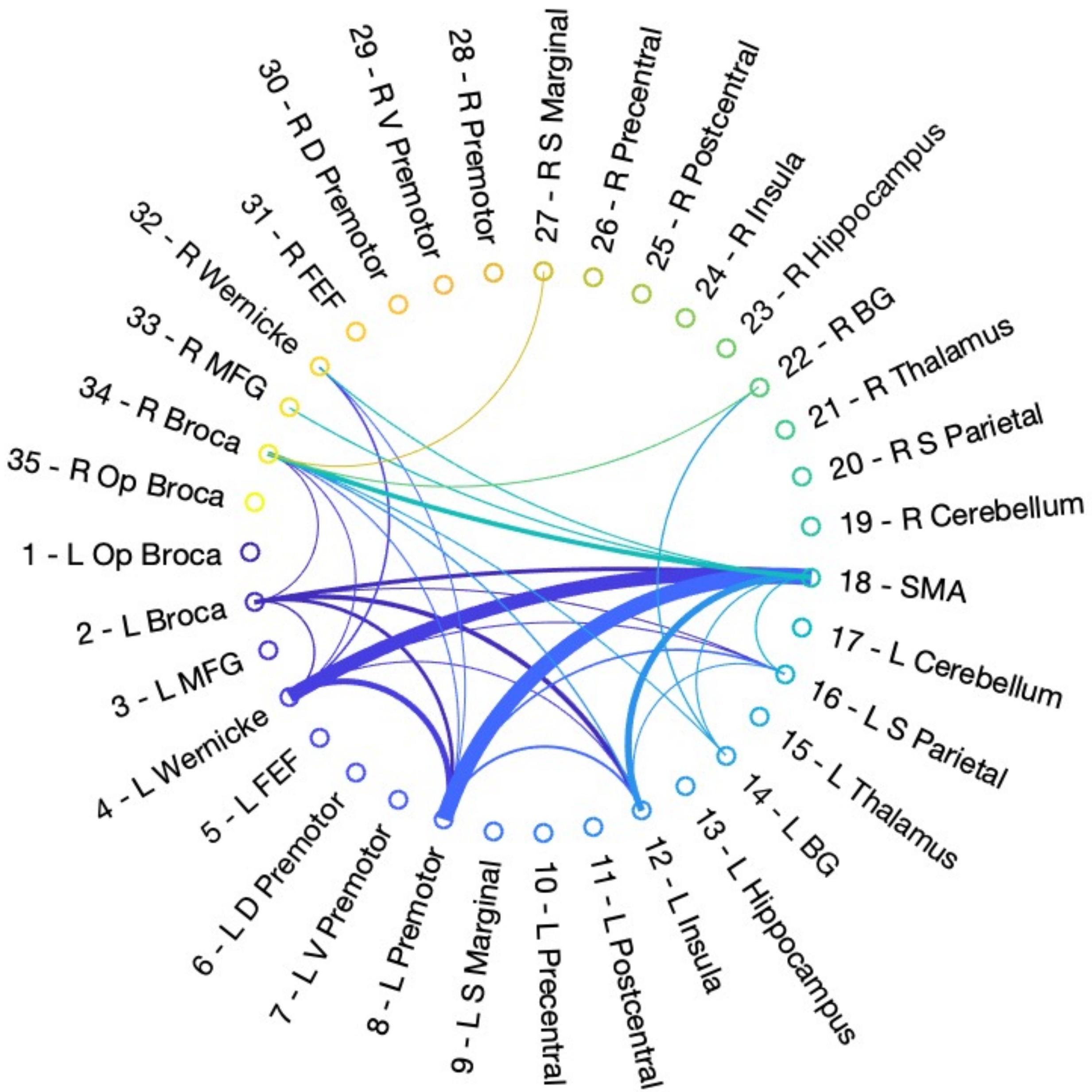

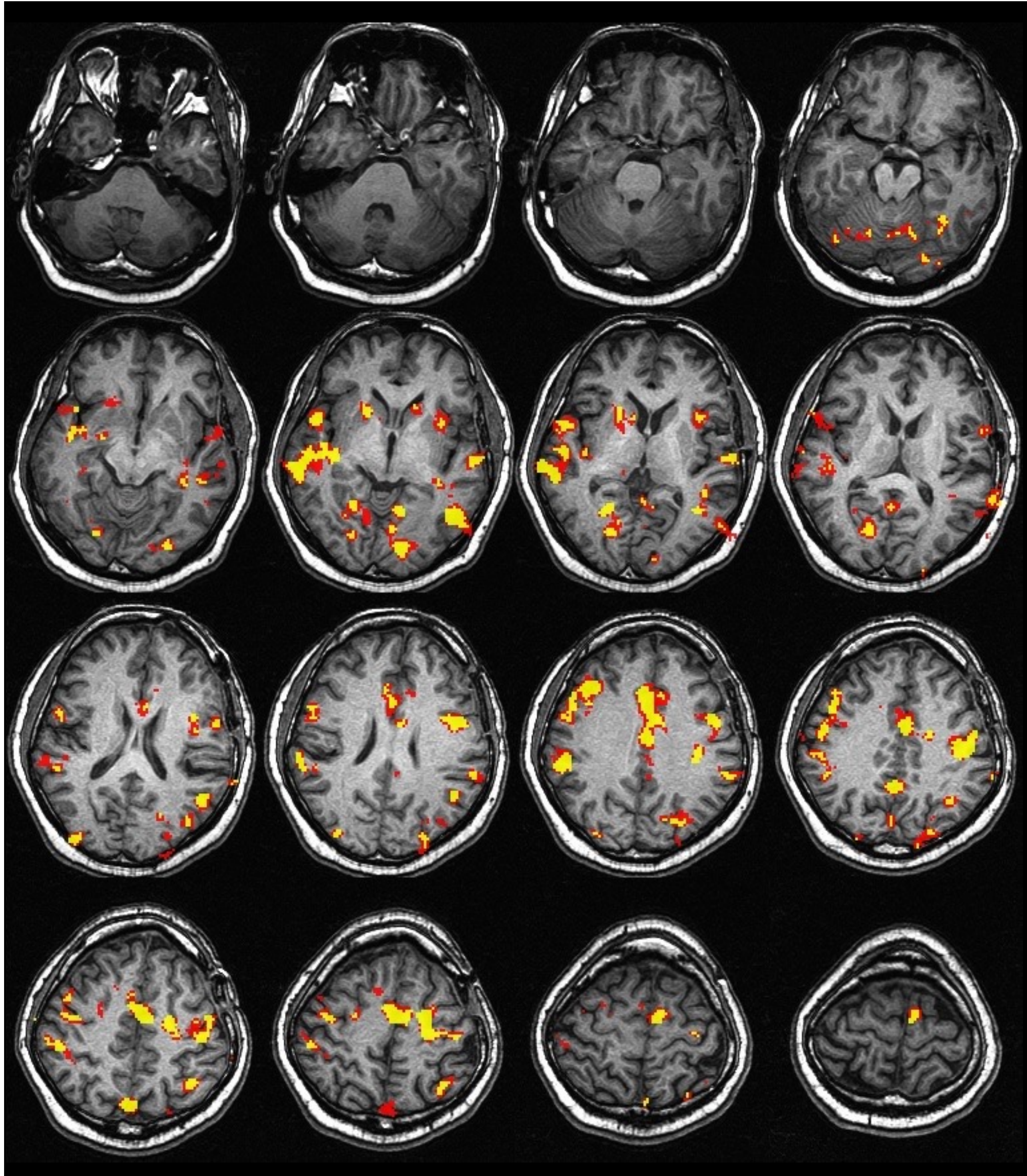

Figure S11. Case3 post-op2.

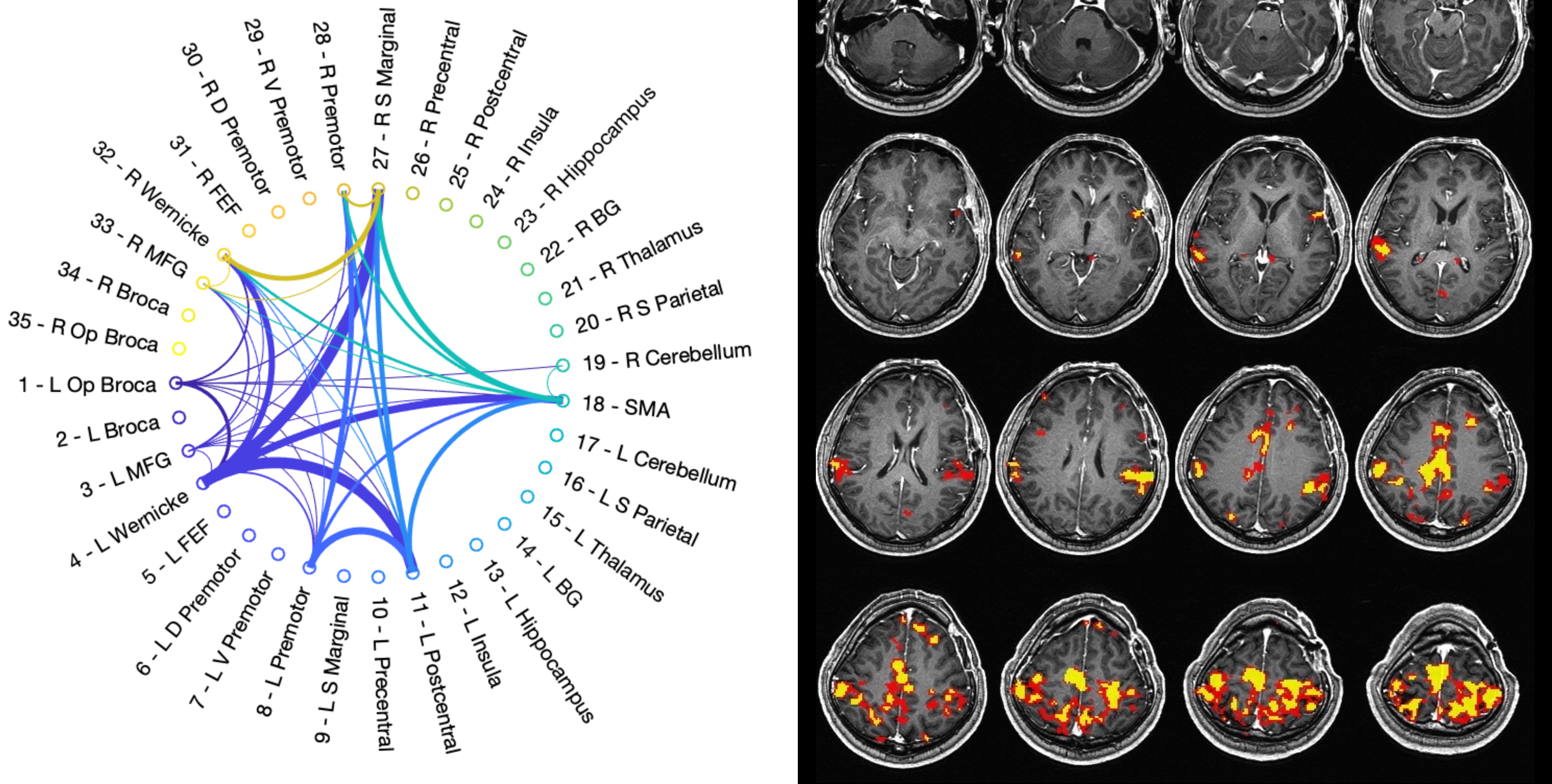

Figure S12. Case3 post-op3.

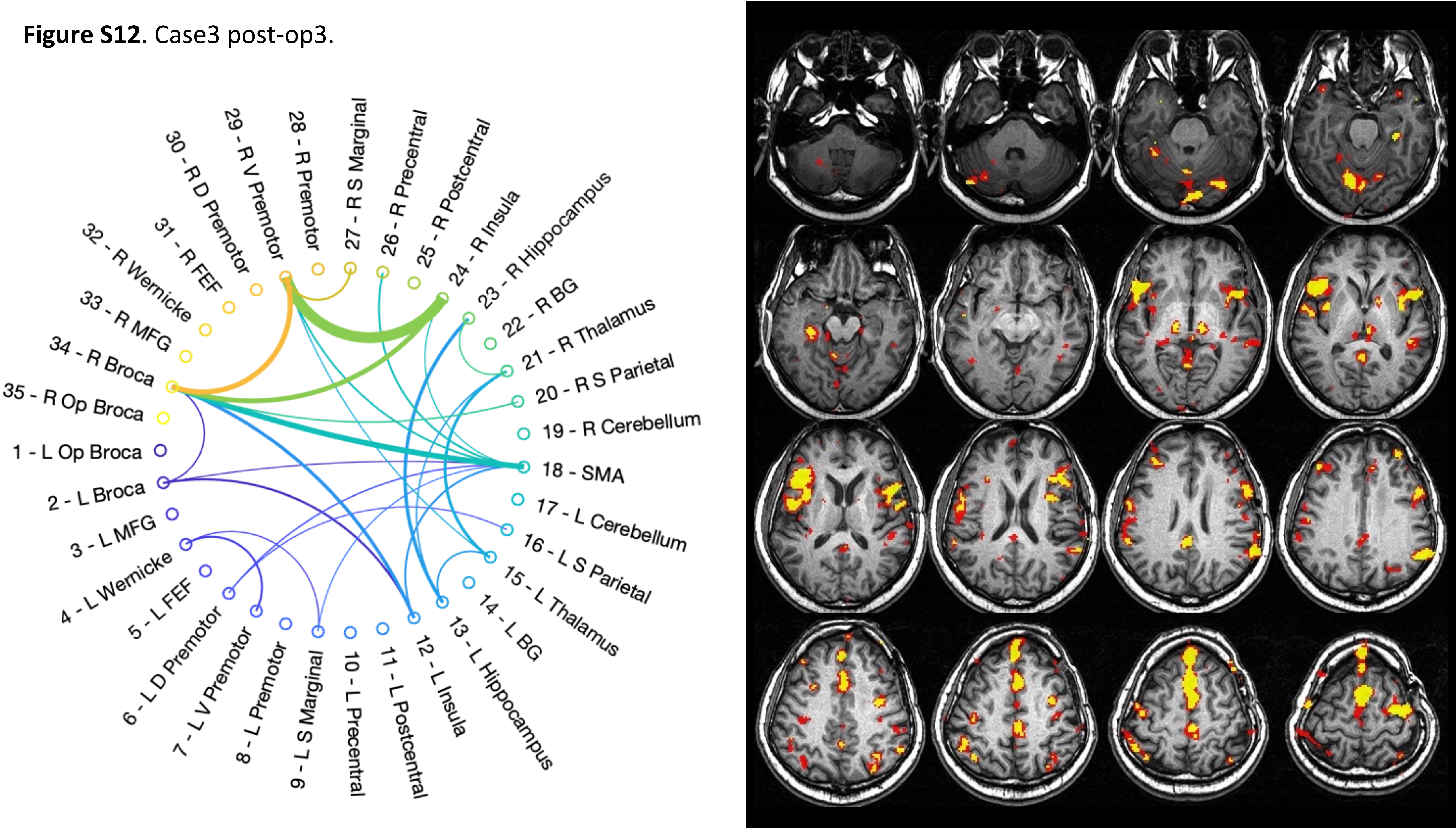

Figure S13. Case4 pre-op.

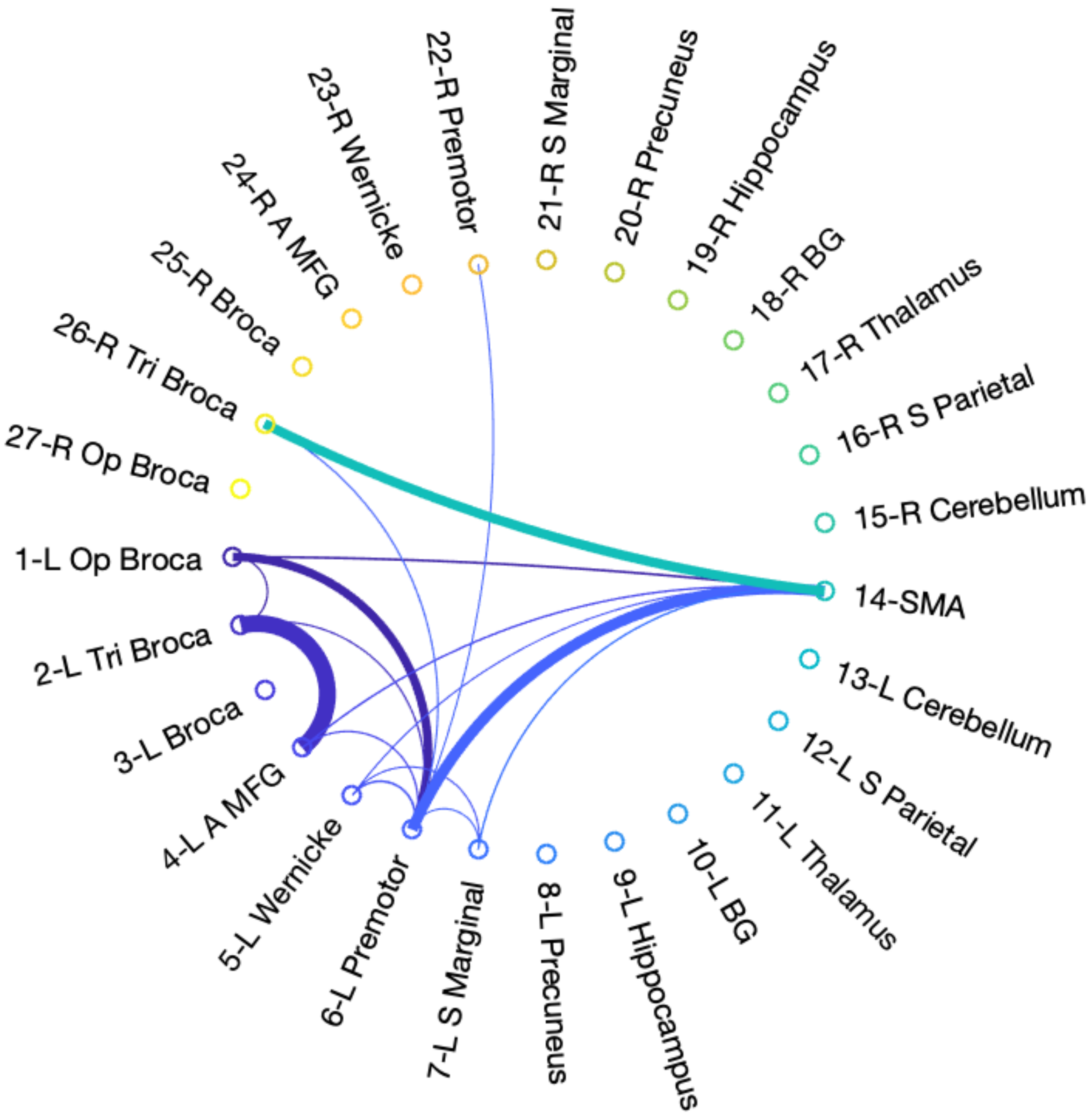

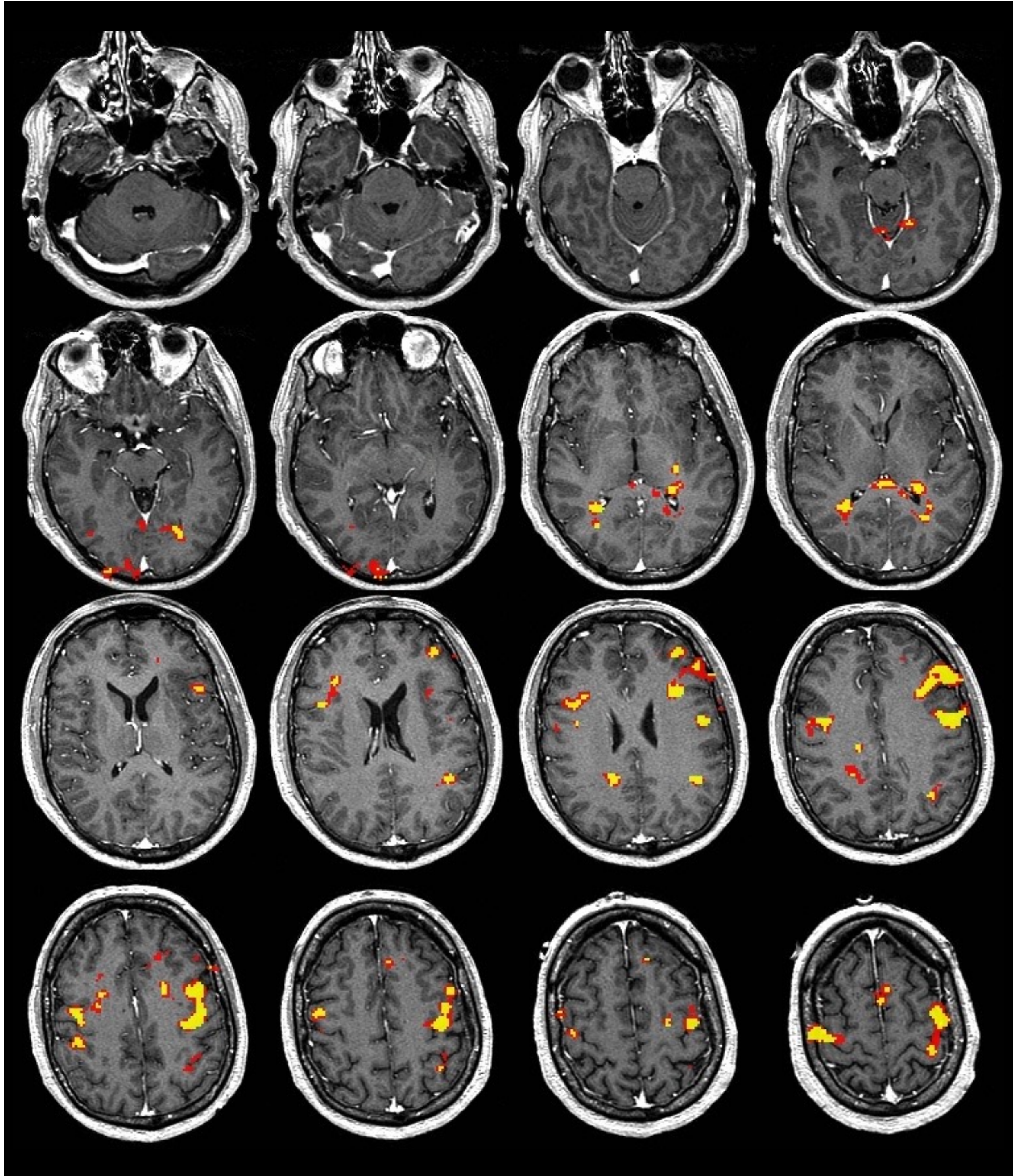

Figure S14. Case4 post-op1.

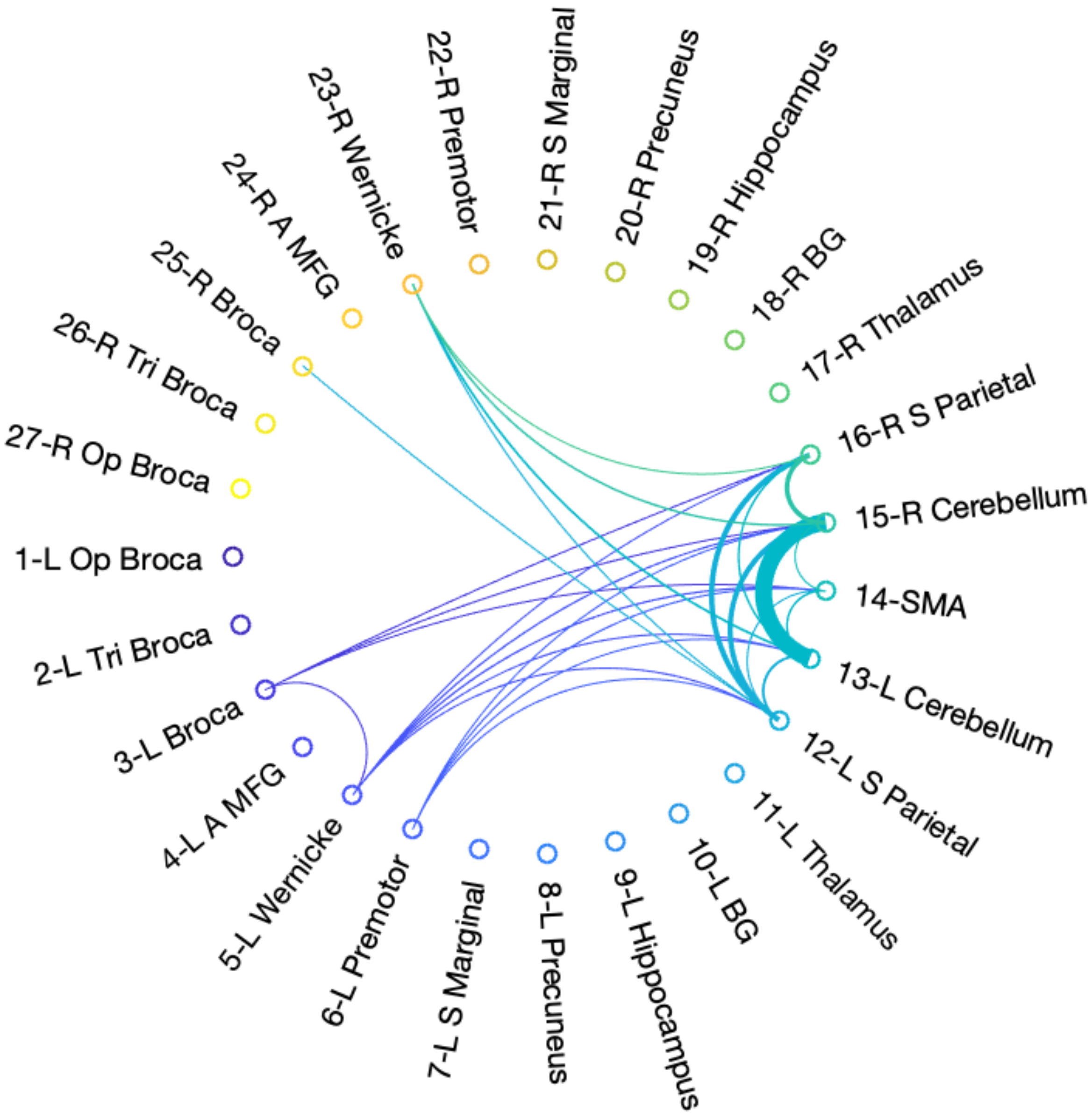

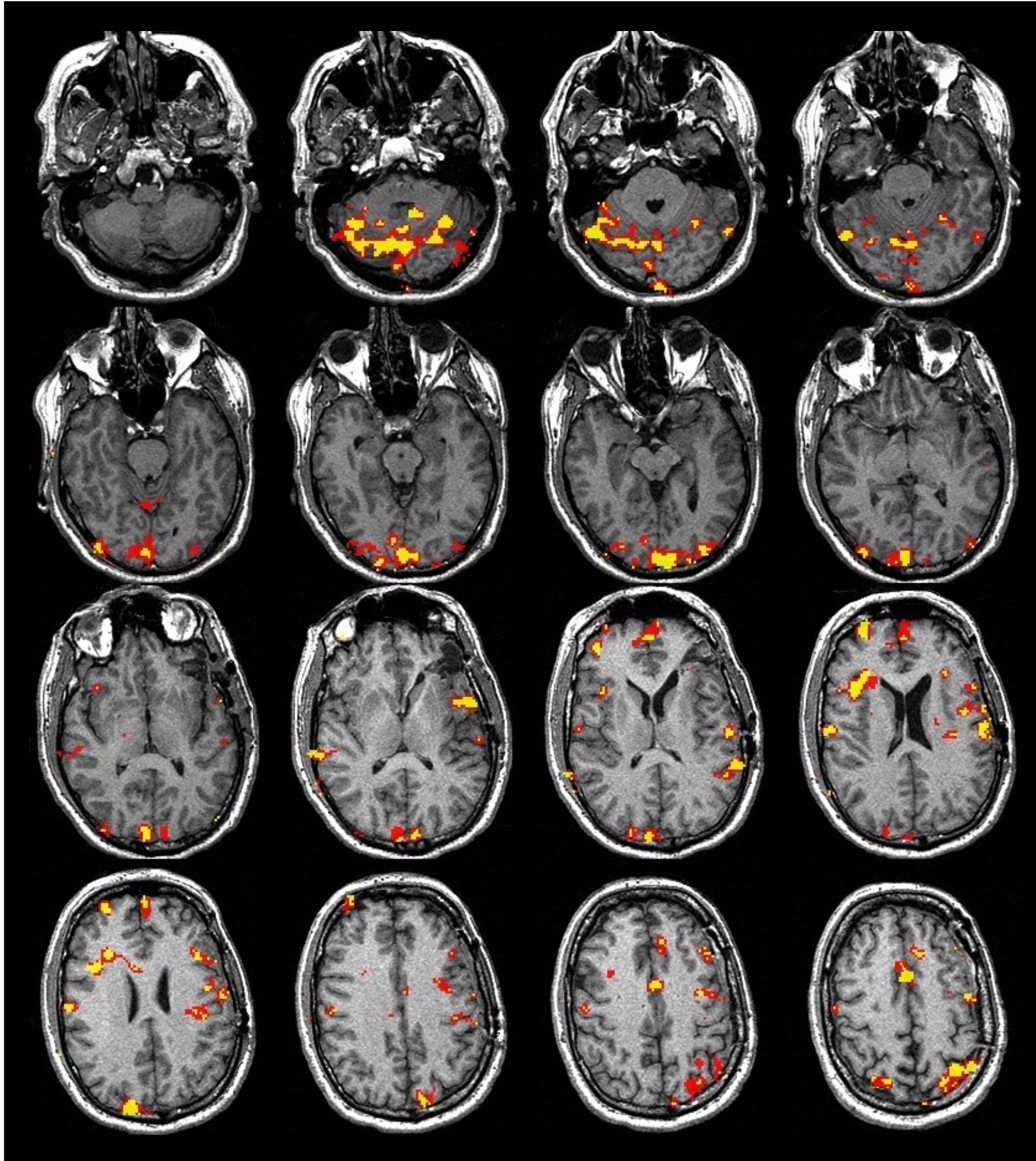

Figure S15. Case4 post-op2.

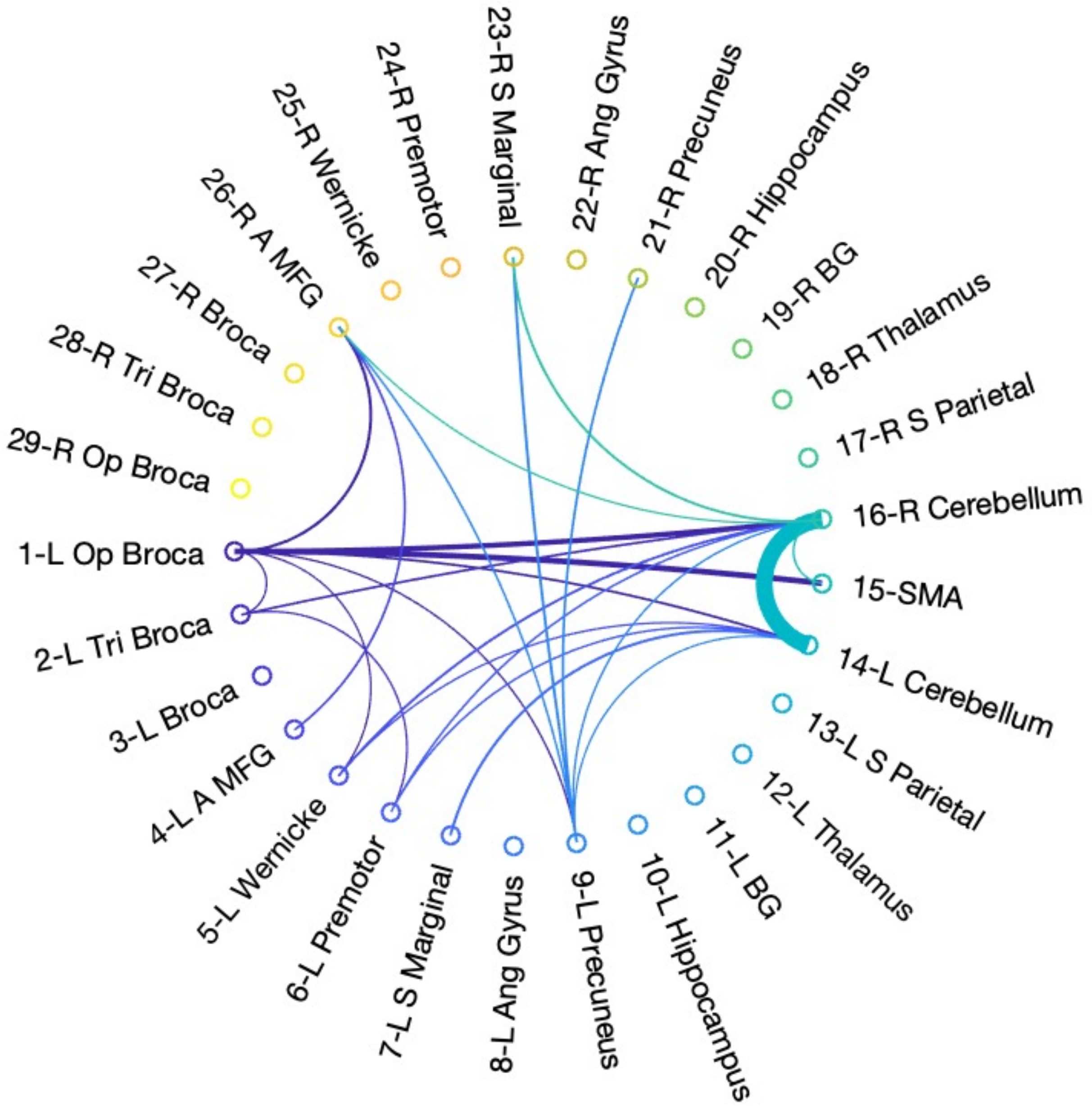

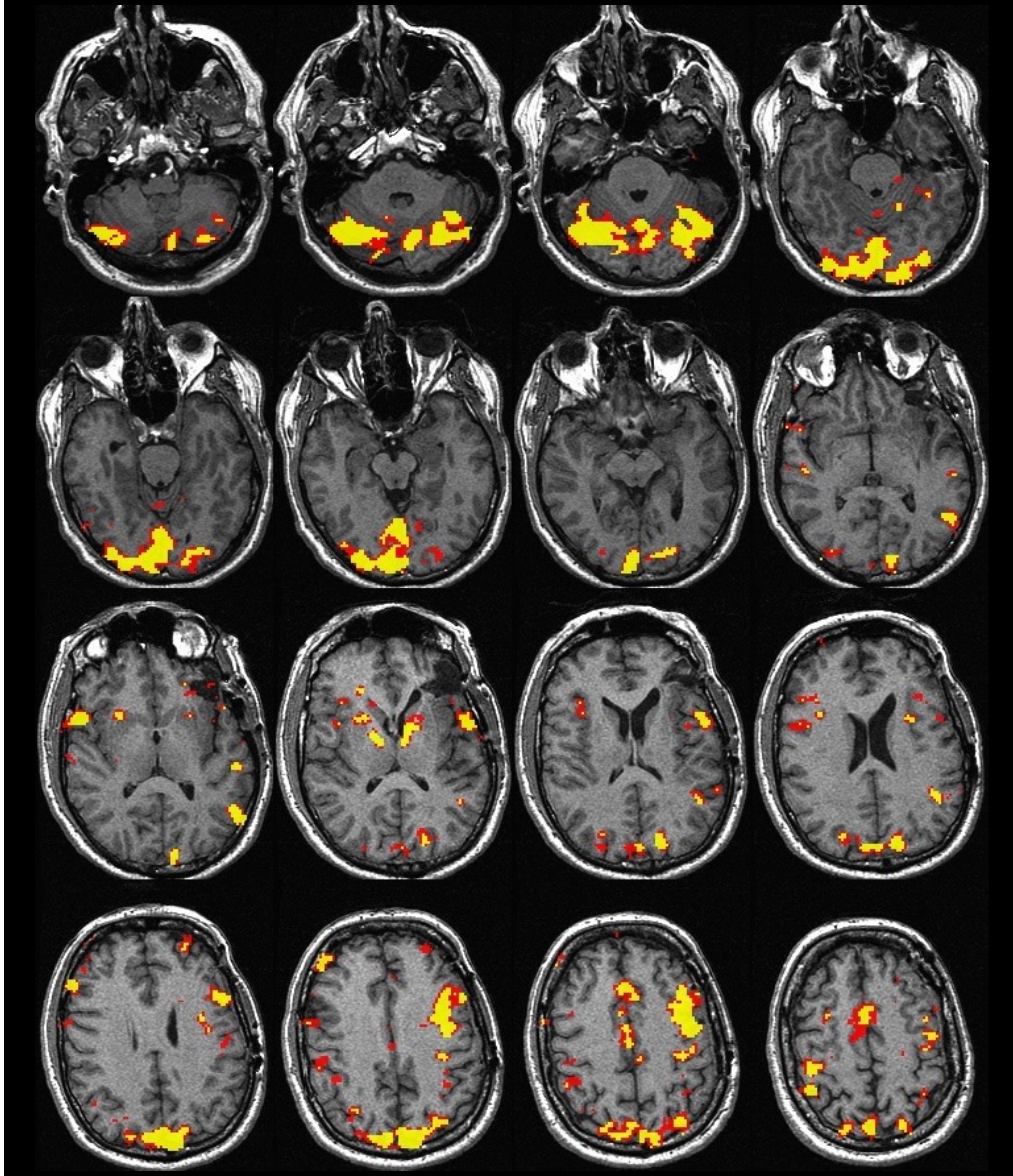

Figure S16. Case4 post-op3.

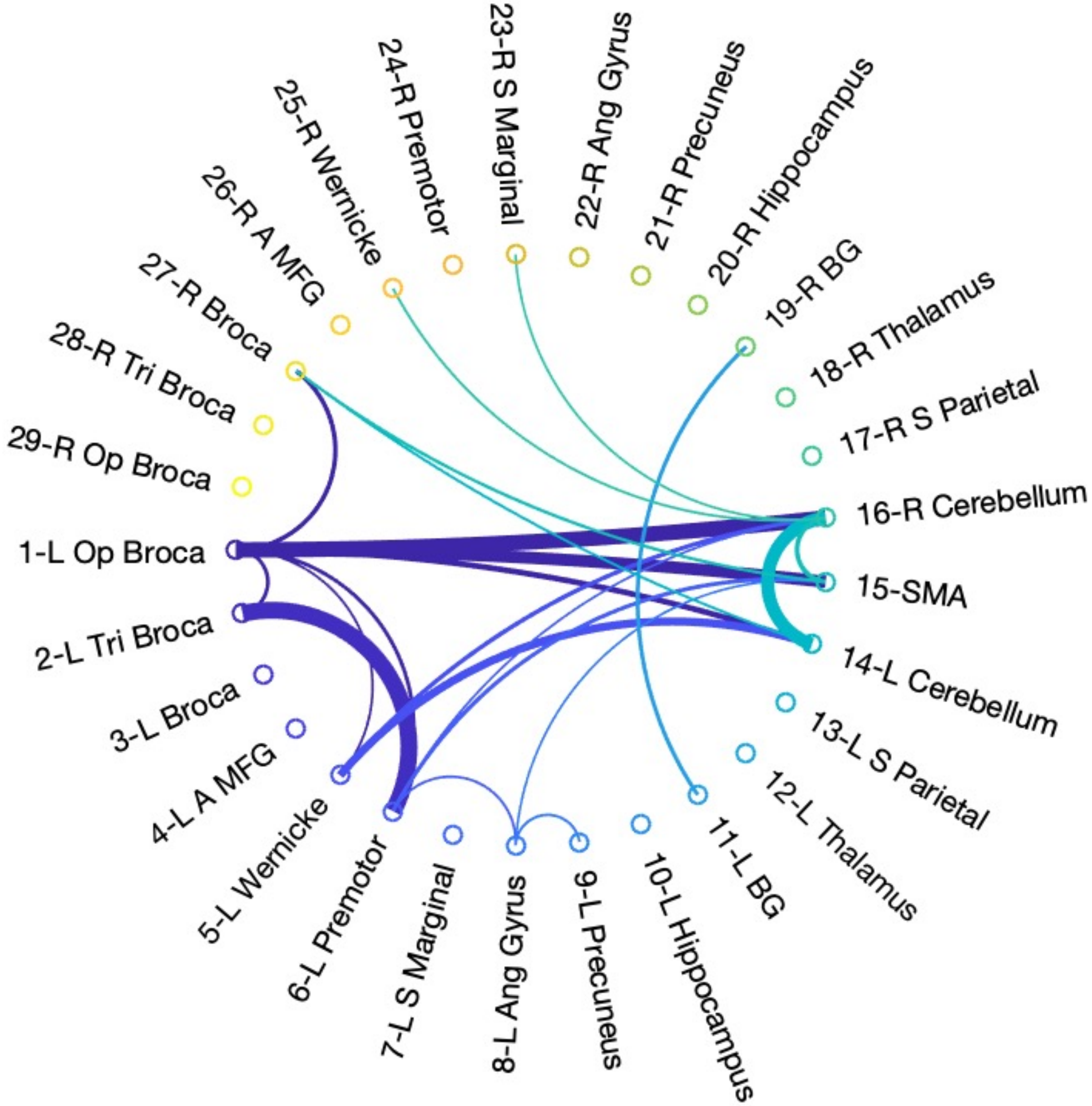

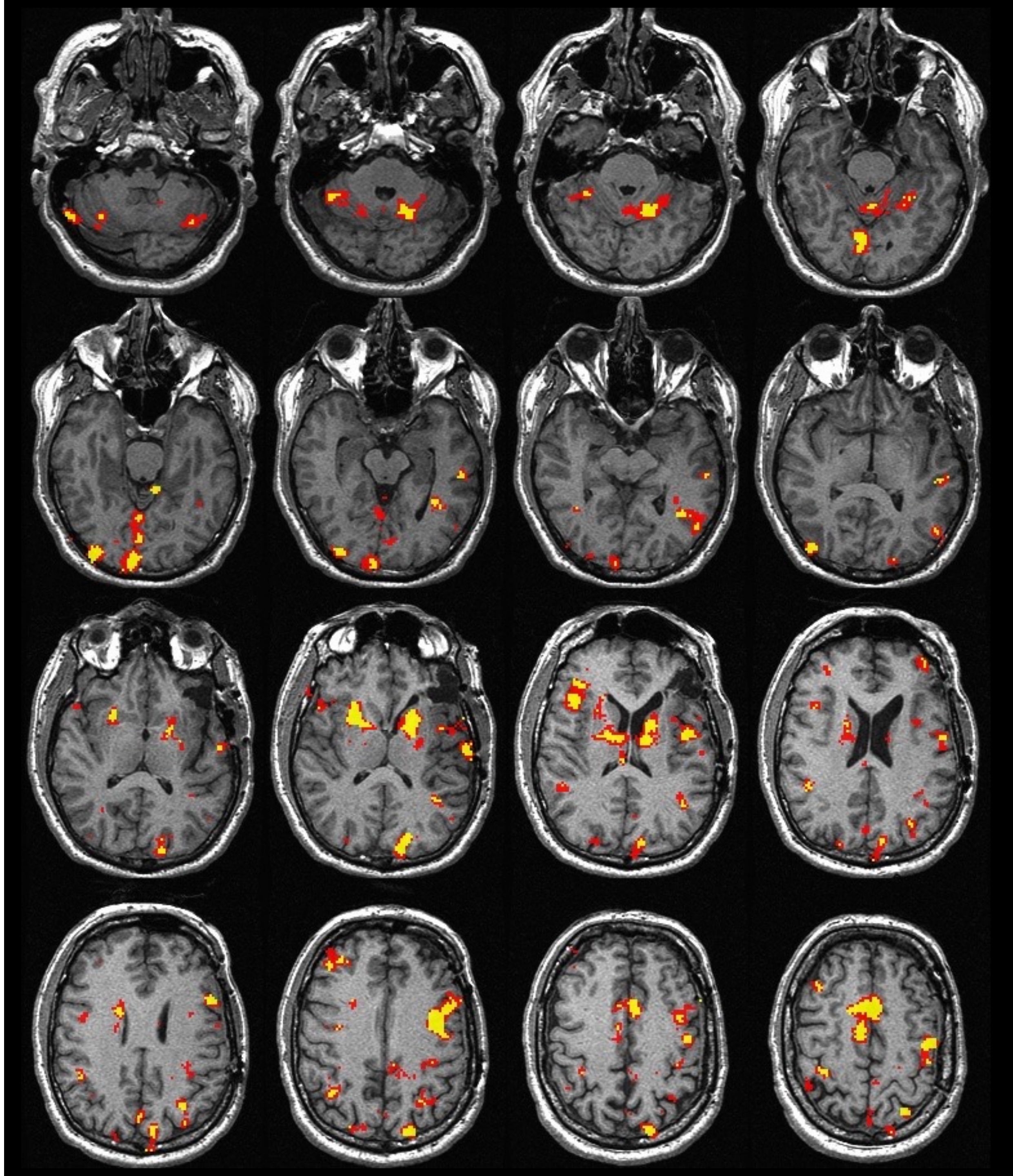

Figure S17. Case5 pre-op.

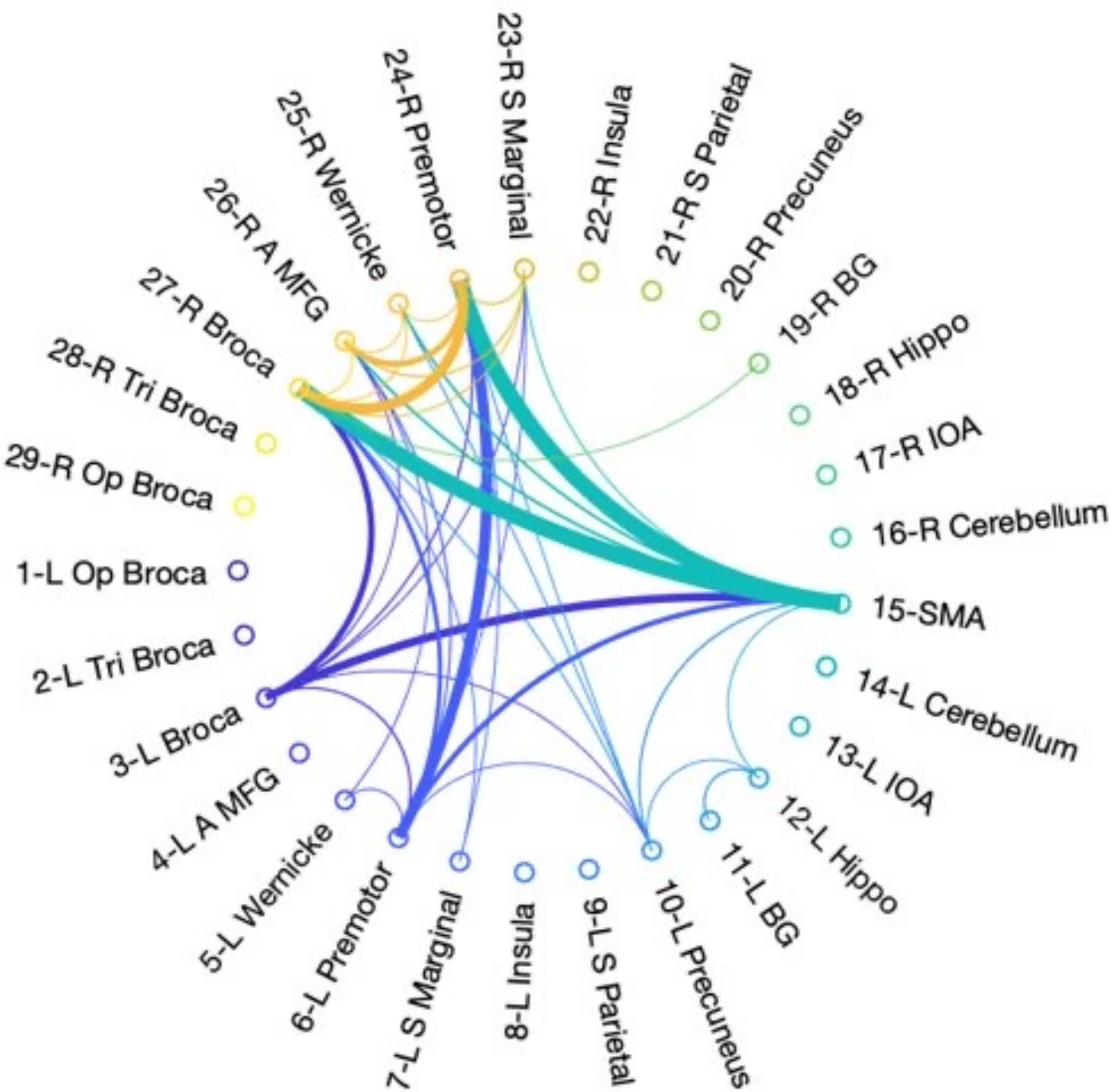

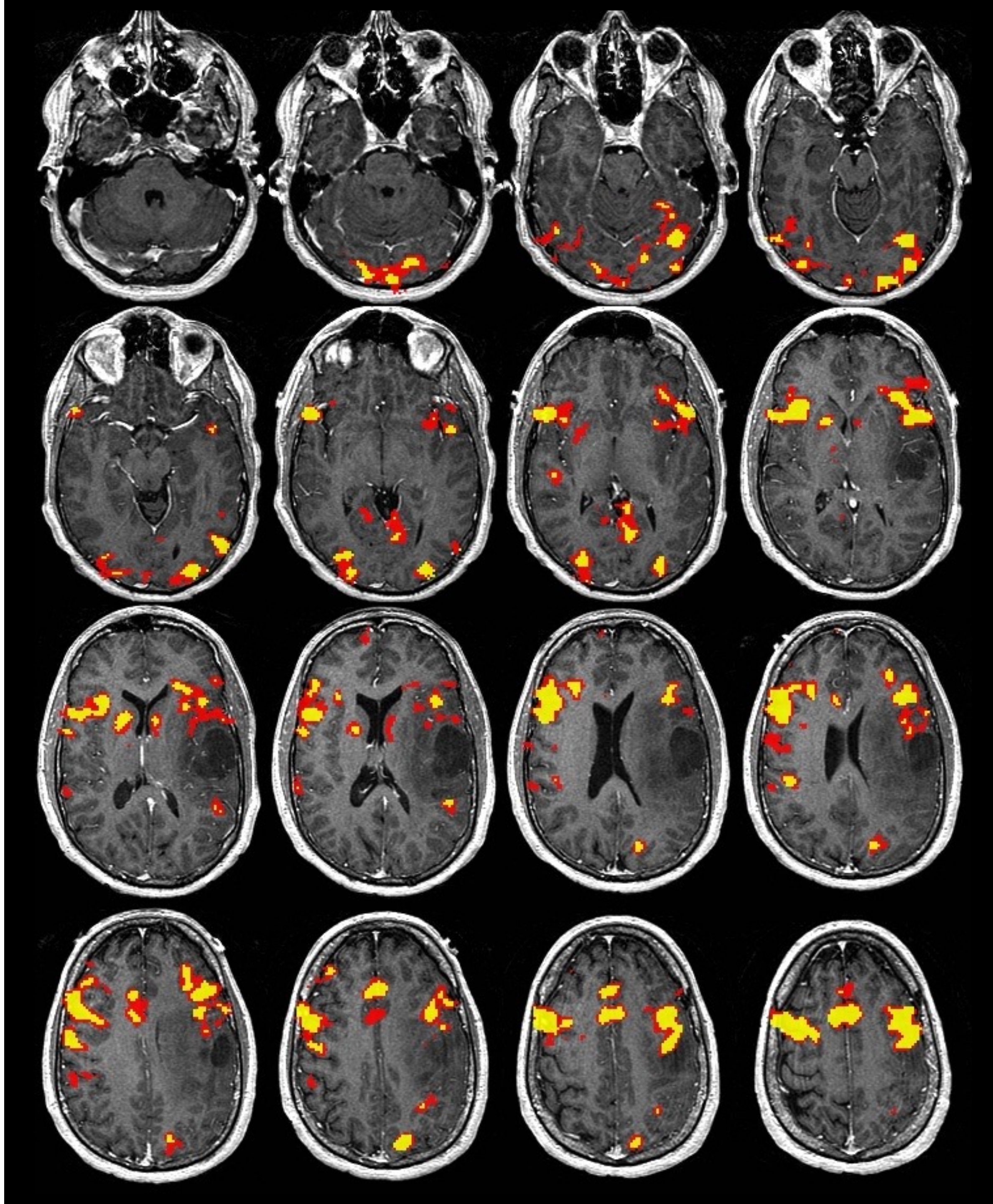

Figure S18. Case5 post-op1.

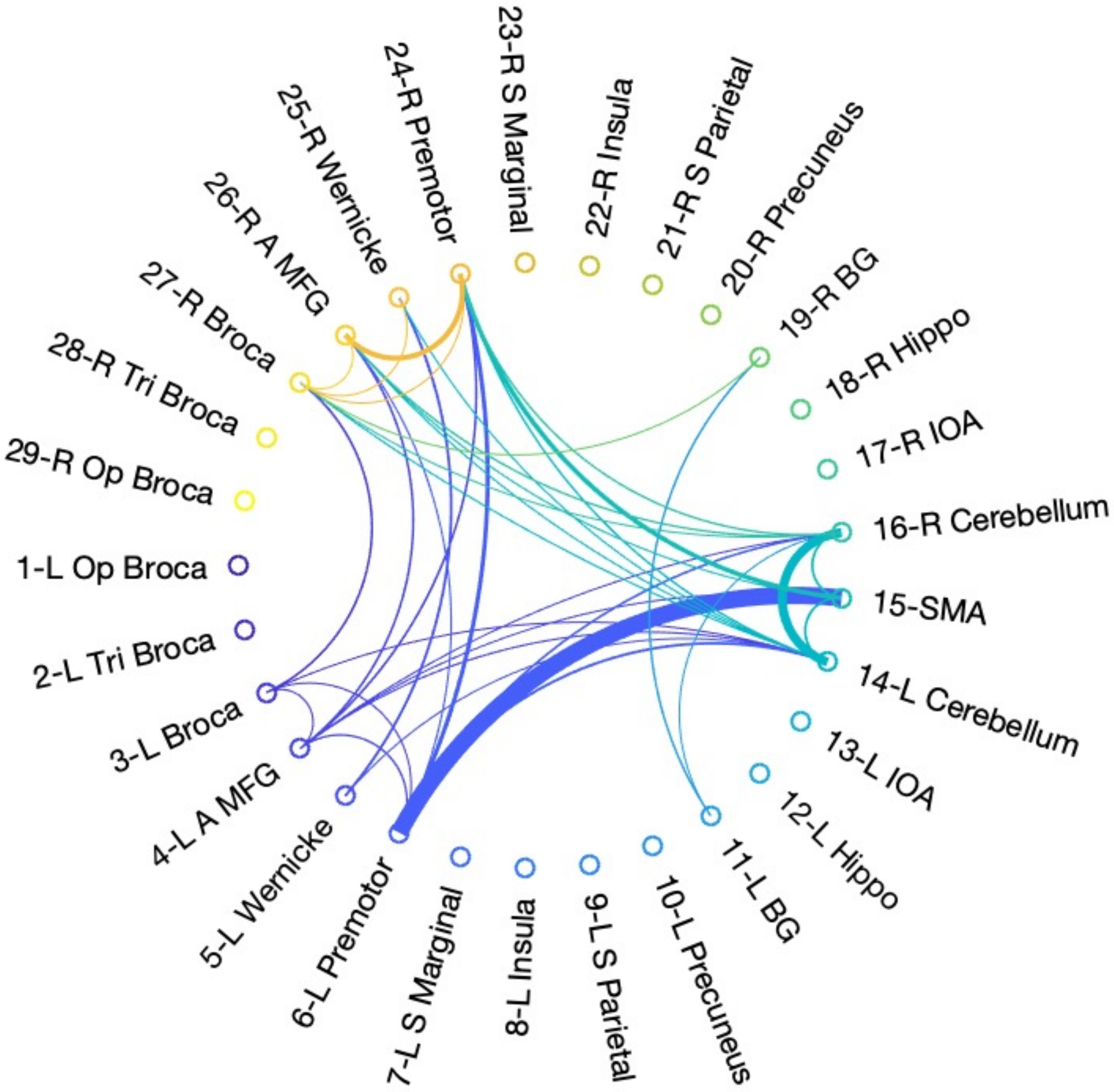

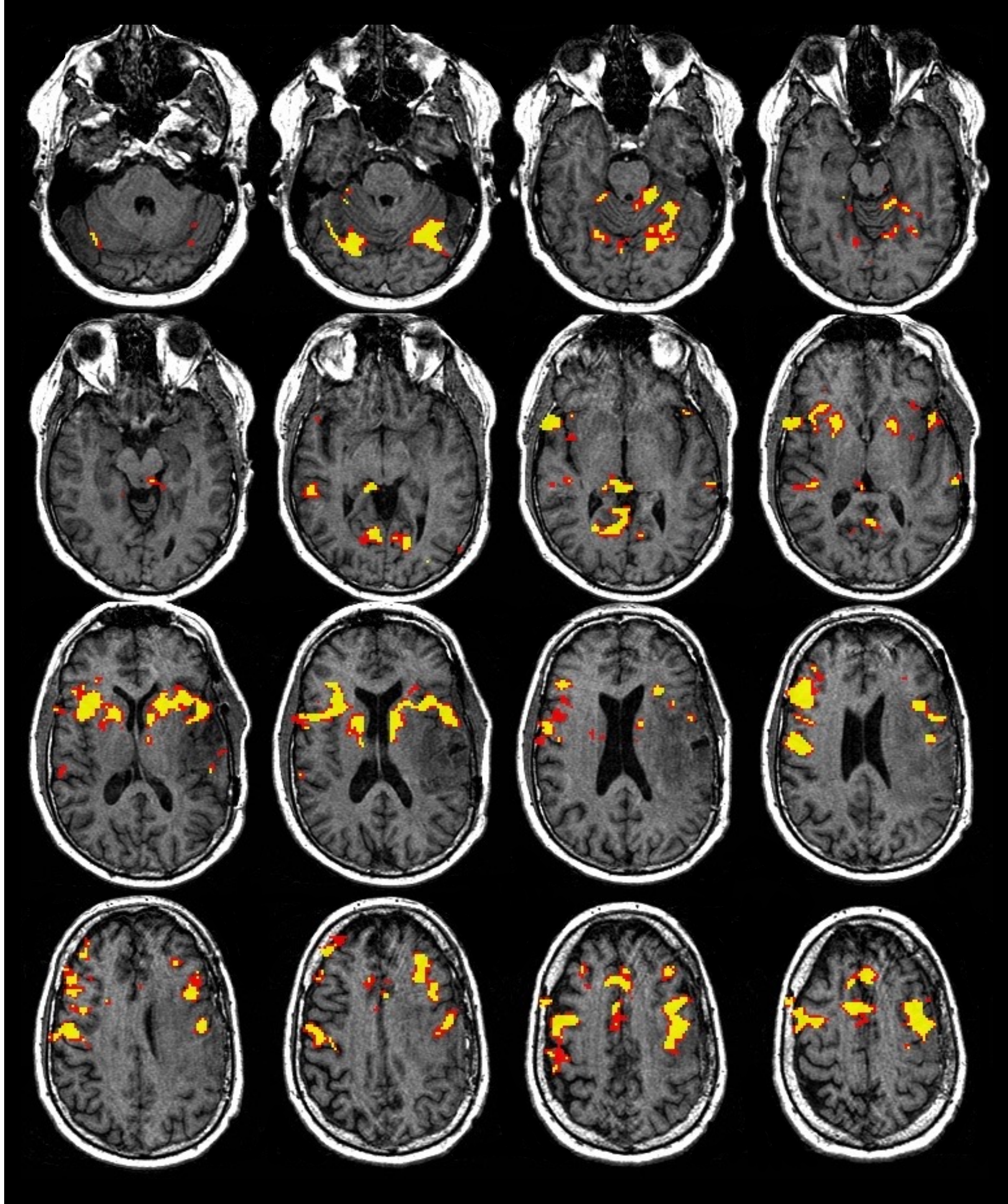

Figure S19. Case5 post-op2.

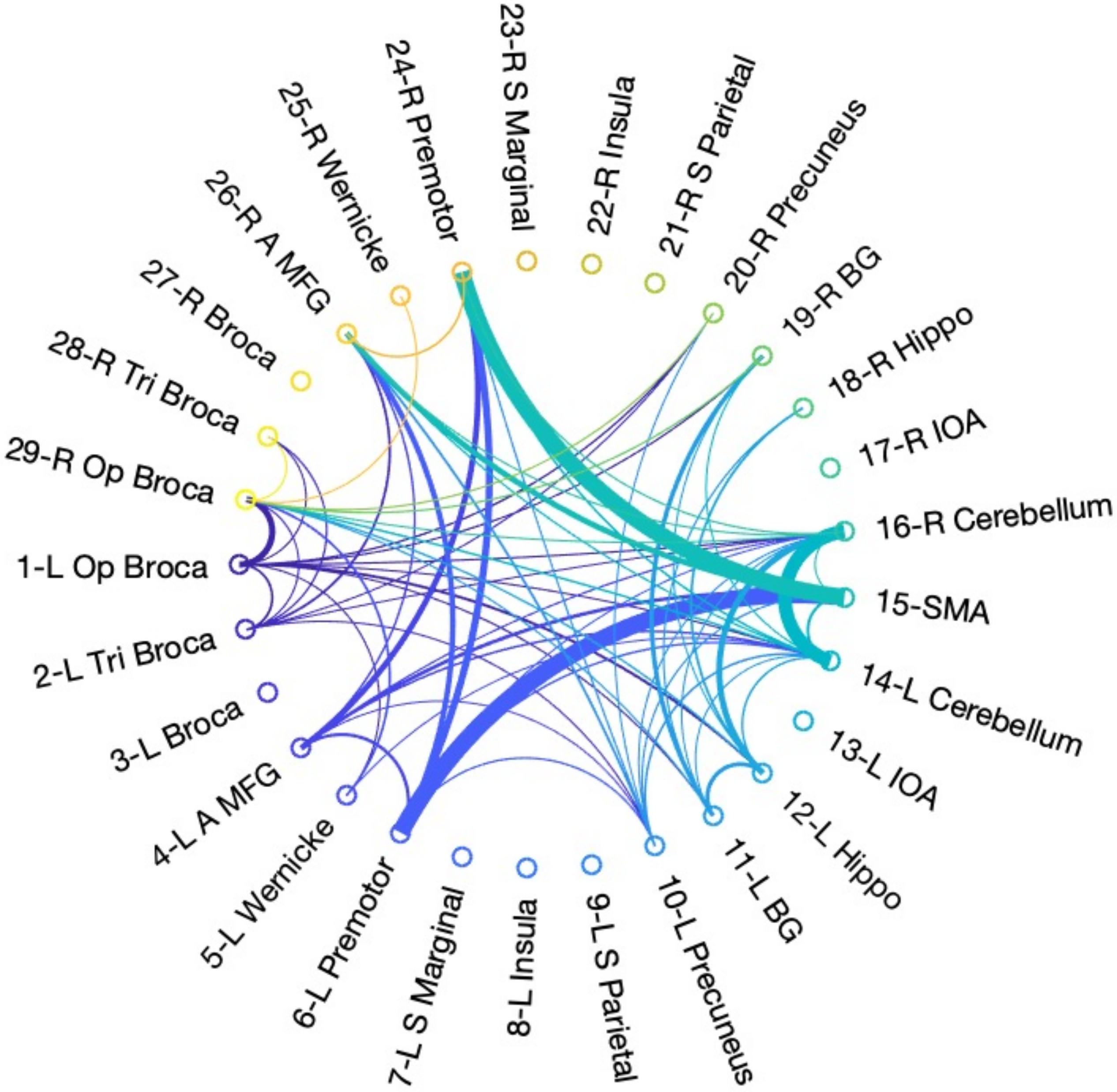

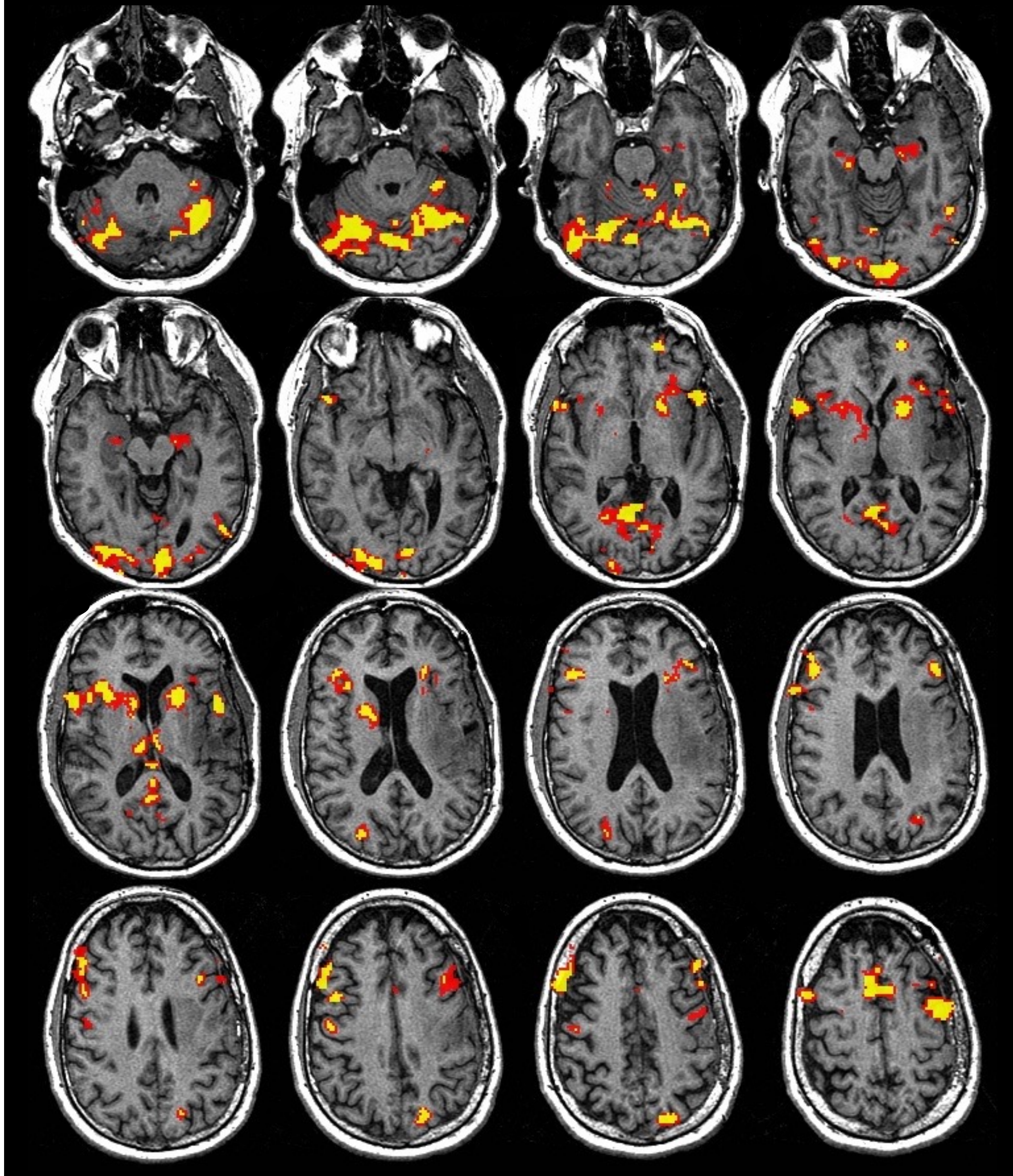

Figure S20. Case5 post-op3.

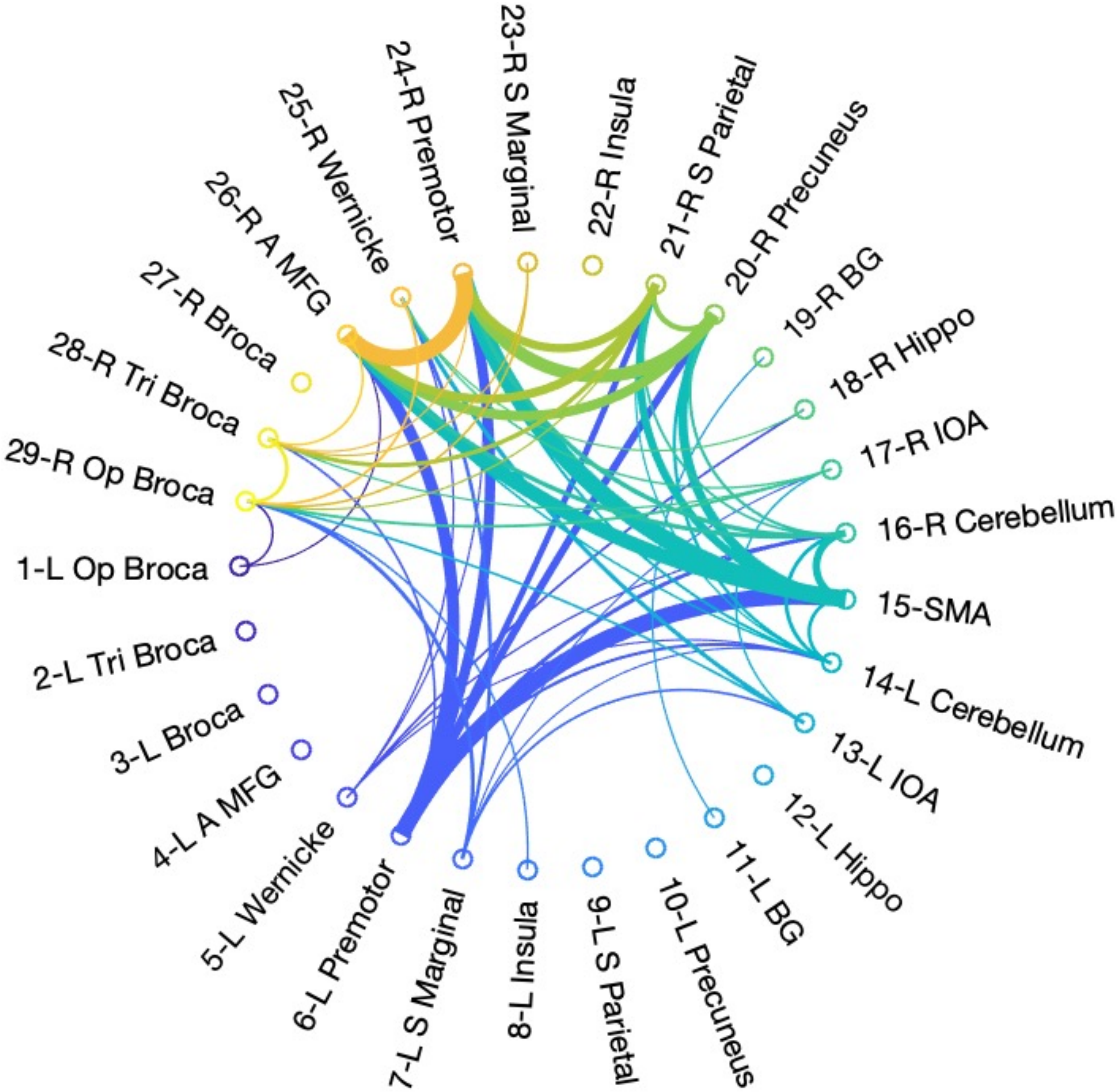

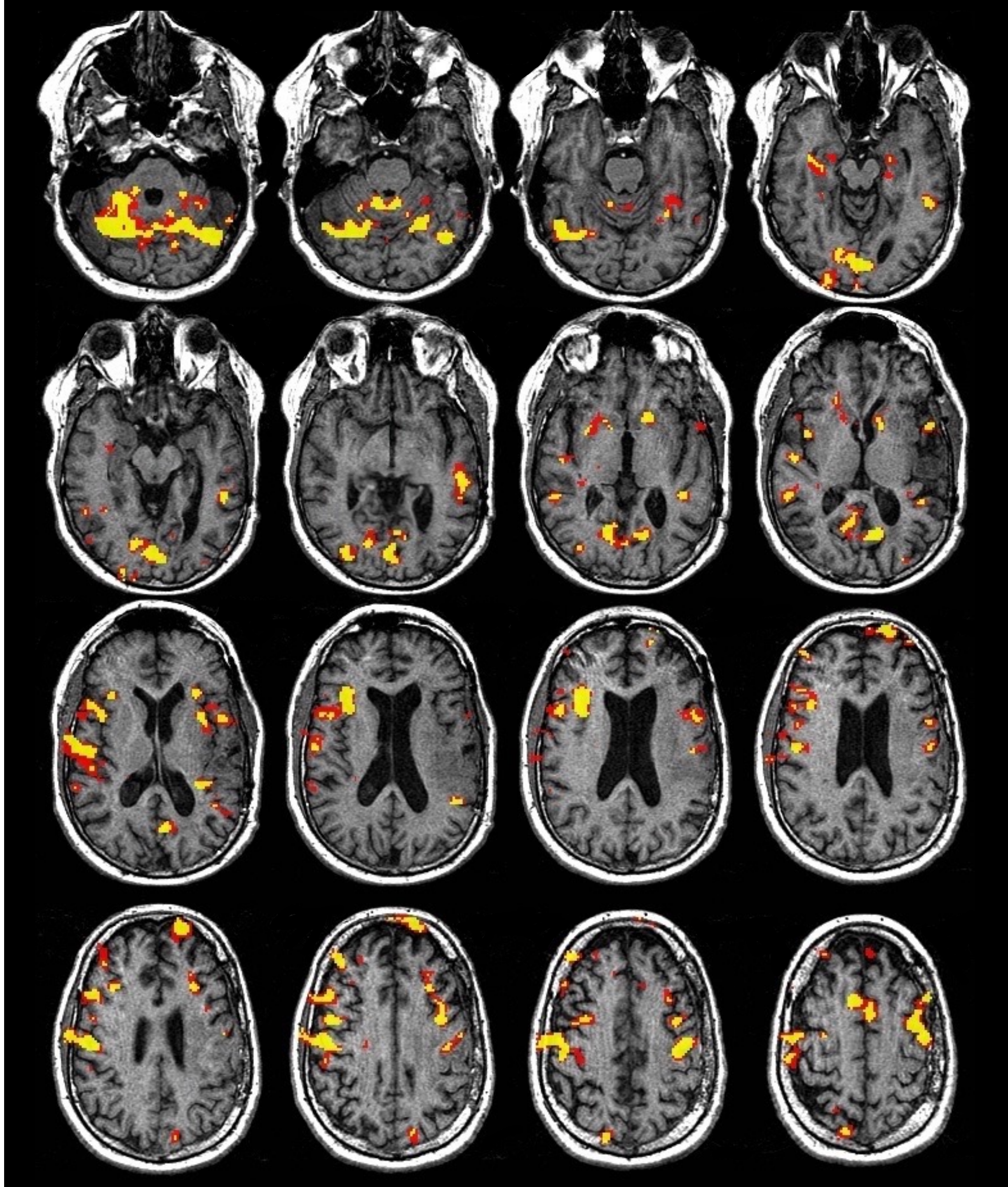

Table S1. Clinical evaluation of speech ability through neurocognitive tests.

Timepoints	BNT raw score (30/60 elements)	BNT Z-score	PVF raw score	PVF Z-score	CF raw score	CF Z-score
CASE 1 Pre-op	NA	NA	NA	NA	NA	NA
CASE 1 Post-op 1	25	0	57	0.89	22	-0.24
CASE 1 Post-op 2	28	0.78	67	1.68	22	-0.24
CASE 1 Post-op 3	NA	NA	NA	NA	NA	NA
CASE 2 Pre-op	8	-3	11	-2.44	8	-2.57
CASE 2 Post-op 1	7	-3	13	-2.3	5	-3
CASE 2 Post-op 2	4	-3	13	-2.3	3	-3
CASE 2 Post-op 3	NA	NA	NA	NA	NA	NA
CASE 3 Pre-op	24	-0.26	29	-1.01	14	-1.67
CASE 3 Post-op 1	28	0.79	34	-0.6	15	-1.43
CASE 3 Post-op 2	28	0.79	24	-1.42	12	-2.15
CASE 3 Post-op 3	27	0.53	28	-1.09	15	-1.43
CASE 4 Pre-op	NA	NA	NA	NA	NA	NA
CASE 4 Post-op 1	29	1.05	45	0.31	18	-0.71
CASE 4 Post-op 2	30	1.32	35	-0.51	20	-0.23
CASE 4 Post-op 3	29	1.05	46	0.39	22	0.25
CASE 5 Pre-op	26	0.26	14	-2.55	21	-0.44
CASE 5 Post-op 1	29	1.05	25	-1.67	23	-0.07
CASE 5 Post-op 2	27	0.53	12	-2.71	24	0.11
CASE 5 Post-op 3	28	0.79	23	-1.83	22	-0.26

BNT=Boston Naming Test; CF=category fluency; NA=non-available; PVF=phonemic verbal fluency

Table S2-6: Functional ROIs.

Here we present supplementary tables including functional ROIs of every patient. Overall, 51 fROIs were identified. Due to intra- and inter-subject variability, which are common in clinical fMRI of brain tumors, not all the areas were active at every timepoint and across patients. Active areas in the left (L) and right (R) hemisphere included: inferior frontal gyrus opercular part (OP BROCA), inferior frontal gyrus triangular part (TRI BROCA), inferior frontal gyrus orbital part (IF ORBITAL), middle frontal gyrus (MFG), middle frontal gyrus anterior division (A MFG), dorsal pre-motor cortex (D PREMOTOR), ventral pre-motor cortex (V PREMOTOR), frontal eye field (FEF), precentral gyrus (PRECENTRAL), post-central gyrus (POSTCENTRAL), Wernicke's area (WERNICKE), supramarginal gyrus (S MARGINAL), angular gyrus (ANG GYRUS), superior parietal lobule (S PARIETAL), precuneus, insula, insular-opercular angle (IOA), hippocampus, thalamus, caudate nucleus (CAUDATE), basal ganglia (BG), visual word form area (VWFA), cerebellum, pre-supplementary motor area (SMA). In some cases, an anatomical subdivision of the functional area was possible, for example when a separate activation was present in the opercular and/or triangular part of the inferior frontal gyrus (Broca's area). In these cases the labeling followed the anatomic subdivision, while in the other cases it followed the general cluster (BROCA).

Table S2 Active clusters for patient 1 at different timepoints (threshold criteria r>0.5; uncorrected $p = 2 \times 10^{-11}$).

CASE 1	T1	T2	T3	T4
**1-L OP BROCA**			x	x
**2-L TRI BROCA**			x	

**3-L BROCA**	x	x		
**4-L IF ORBITAL**	x			
**5-L A MFG**			x	
**6-L MFG**	x	x		
**7-L D PREMOTOR**	x	x		
**8-L V PREMOTOR**	x	x		
**9-L PREMOTOR**			x	x
**10-L FEF**				
**11-L PRECENTRAL**				
**12-L POSTCENTRAL**				
**13-L WERNICKE**	x	x	x	x
**14-L S MARGINAL**	x		x	x
**15-L ANG GYRUS**				x
**16-L S PARIETAL**				
**17-L PRECUNEUS**			x	x
**18-L INSULA**				
**19-L IOA**				
**20-L HIPPOCAMPUS**				
**21-L THALAMUS**				
**22-L CAUDATE**	x	x		
**23-L BG**			x	x
**24-L VWFA**		x		
**25-L CEREBELLUM**			x	x
**26-SMA**	x	x	x	x
**27-R CEREBELLUM**		x	x	x
**28-R VWFA**				
**29-R BG**			x	

**30-R CAUDATE**		x		
**31-R THALAMUS**				
**32-R HIPPOCAMPUS**				
**33-R-IOA**				
**34-R INSULA**				x
**35-R PRECUNEUS**			x	
**36-R S PARIETAL**				
**37-R ANG GYRUS**				
**38-R S MARGINAL**				x
**39-R WERNICKE**		x	x	x
**40-R POSTCENTRAL**				
**41-R PRECENTRAL**				
**42-R FEF**				
**43-R PREMOTOR**			x	
**44-R V PREMOTOR**				
**45-R D PREMOTOR**				
**46-R MFG**				
**47-R A MFG**			x	x
**48-R IF ORBITAL**	x			
**49-R BROCA**		x		
**50-R TRI BROCA**			x	x
**51-R OP BROCA**			x	

Table S3 Active clusters for patient 2 at different timepoints (threshold criteria $r>0.5$; uncorrected $p = 2 \times 10^{-11}$).

**CASE 2**	**T1**	**T2**	**T3**	**T4**

**1-L OP BROCA**		x		
**2-L TRI BROCA**		x	x	x
**3-L BROCA**	x		x	
**4-L IF ORBITAL**				
**5-L A MFG**		x		x
**6-L MFG**	x		x	
**7-L D PREMOTOR**			x	
**8-L V PREMOTOR**			x	
**9-L PREMOTOR**	x	x		x
**10-L FEF**				
**11-L PRECENTRAL**				
**12-L POSTCENTRAL**				
**13-L WERNICKE**	x	x	x	x
**14-L S MARGINAL**			x	
**15-L ANG GYRUS**	x	x	x	
**16-L S PARIETAL**			x	
**17-L PRECUNEUS**				
**18-L INSULA**				
**19-L IOA**				
**20-L HIPPOCAMPUS**				
**21-L THALAMUS**	x			
**22-L CAUDATE**				
**23-L BG**				x
**24-L VWFA**				
**25-L CEREBELLUM**	x	x	x	x
**26-SMA**	x	x	x	x
**27-R CEREBELLUM**	x	x		x
**28-R VWFA**				

**29-R BG**	x	x		x
**30-R CAUDATE**				
**31-R THALAMUS**	x	x		
**32-R HIPPOCAMPUS**		x		x
**33-R-IOA**				
**34-R INSULA**				
**35-R PRECUNEUS**				
**36-R S PARIETAL**				
**37-R ANG GYRUS**	x			
**38-R S MARGINAL**			x	
**39-R WERNICKE**			x	
**40-R POSTCENTRAL**				
**41-R PRECENTRAL**				
**42-R FEF**				
**43-R PREMOTOR**	x	x		
**44-R V PREMOTOR**				
**45-R D PREMOTOR**			x	
**46-R MFG**	x		x	
**47-R A MFG**		x		x
**48-R IF ORBITAL**				x
**49-R BROCA**	x	x		
**50-R TRI BROCA**				x
**51-R OP BROCA**			x	

Table S4 Active clusters for patient 3 at different timepoints (threshold criteria $r>0.5$; uncorrected $p = 2 \times 10^{-11}$).

**CASE 3**	**T1**	**T2**	**T3**	**T4**

**1-L OP BROCA**			x	
**2-L TRI BROCA**				
**3-L BROCA**	x	x		x
**4-L IF ORBITAL**				
**5-L A MFG**				
**6-L MFG**			x	x
**7-L D PREMOTOR**	x			x
**8-L V PREMOTOR**	x			x
**9-L PREMOTOR**		x	x	
**10-L FEF**				
**11-L PRECENTRAL**				
**12-L POSTCENTRAL**			x	
**13-L WERNICKE**	x	x	x	x
**14-L S MARGINAL**				x
**15-L ANG GYRUS**				
**16-L S PARIETAL**	x	x		x
**17-L PRECUNEUS**				
**18-L INSULA**	x	x		x
**19-L IOA**				
**20-L HIPPOCAMPUS**				x
**21-L THALAMUS**	x			x
**22-L CAUDATE**				
**23-L BG**	x	x		
**24-L VWFA**				
**25-L CEREBELLUM**				x
**26-SMA**	x	x	x	x
**27-R CEREBELLUM**			x	x
**28-R VWFA**				

**29-R BG**		x		
**30-R CAUDATE**				
**31-R THALAMUS**				x
**32-R HIPPOCAMPUS**				x
**33-R-IOA**				
**34-R INSULA**				x
**35-R PRECUNEUS**				
**36-R S PARIETAL**	x			x
**37-R ANG GYRUS**				
**38-R S MARGINAL**		x	x	x
**39-R WERNICKE**		x	x	
**40-R POSTCENTRAL**				
**41-R PRECENTRAL**				x
**42-R FEF**				
**43-R PREMOTOR**			x	
**44-R V PREMOTOR**				x
**45-R D PREMOTOR**	x			
**46-R MFG**		x	x	x
**47-R A MFG**				
**48-R IF ORBITAL**				
**49-R BROCA**		x		x
**50-R TRI BROCA**				
**51-R OP BROCA**				

Table S5 Active clusters for patient 4 at different timepoints (threshold criteria $r>0.5$; uncorrected $p = 2 \times 10^{-11}$).

**CASE 4**	**T1**	**T2**	**T3**	**T4**

**1-L OP BROCA**	x		x	x
**2-L TRI BROCA**	x		x	x
**3-L BROCA**		x		
**4-L IF ORBITAL**				
**5-L A MFG**	x		x	x
**6-L MFG**				
**7-L D PREMOTOR**				
**8-L V PREMOTOR**				
**9-L PREMOTOR**	x	x	x	x
**10-L FEF**				
**11-L PRECENTRAL**				
**12-L POSTCENTRAL**				
**13-L WERNICKE**	x	x	x	x
**14-L S MARGINAL**	x		x	
**15-L ANG GYRUS**				x
**16-L S PARIETAL**		x		
**17-L PRECUNEUS**			x	x
**18-L INSULA**				
**19-L IOA**				
**20-L HIPPOCAMPUS**			x	
**21-L THALAMUS**				
**22-L CAUDATE**				
**23-L BG**			x	x
**24-L VWFA**				
**25-L CEREBELLUM**		x	x	x
**26-SMA**	x	x	x	x
**27-R CEREBELLUM**		x	x	x
**28-R VWFA**				

**29-R BG**			x	x
**30-R CAUDATE**				
**31-R THALAMUS**				
**32-R HIPPOCAMPUS**			x	
**33-R-IOA**				
**34-R INSULA**				
**35-R PRECUNEUS**			x	x
**36-R S PARIETAL**		x		
**37-R ANG GYRUS**				
**38-R S MARGINAL**			x	x
**39-R WERNICKE**		x		x
**40-R POSTCENTRAL**				
**41-R PRECENTRAL**				
**42-R FEF**				
**43-R PREMOTOR**	x			
**44-R V PREMOTOR**				
**45-R D PREMOTOR**				
**46-R MFG**				
**47-R A MFG**			x	x
**48-R IF ORBITAL**				
**49-R BROCA**		x		x
**50-R TRI BROCA**	x		x	
**51-R OP BROCA**			x	

Table S6 Active clusters for patient 5 at different timepoints (threshold criteria $r>0.5$; uncorrected $p = 2 \times 10^{-11}$).

CASE 5	T1	T2	T3	T4
**1-L OP BROCA**			x	x
**2-L TRI BROCA**			x	
**3-L BROCA**	x	x		
**4-L IF ORBITAL**				
**5-L A MFG**		x	x	
**6-L MFG**				
**7-L D PREMOTOR**				
**8-L V PREMOTOR**				
**9-L PREMOTOR**	x	x	x	x
**10-L FEF**				
**11-L PRECENTRAL**				
**12-L POSTCENTRAL**				
**13-L WERNICKE**	x	x	x	x
**14-L S MARGINAL**	x			x
**15-L ANG GYRUS**				
**16-L S PARIETAL**				
**17-L PRECUNEUS**	x		x	
**18-L INSULA**				x
**19-L IOA**				x
**20-L HIPPOCAMPUS**	x		x	
**21-L THALAMUS**				
**22-L CAUDATE**				
**23-L BG**	x	x	x	x
**24-L VWFA**				
**25-L CEREBELLUM**			x	x
**26-SMA**	x	x	x	x
**27-R CEREBELLUM**			x	x

**28-R VWFA**				
**29-R BG**	x	x	x	x
**30-R CAUDATE**				
**31-R THALAMUS**				
**32-R HIPPOCAMPUS**			x	x
**33-R-IOA**				x
**34-R INSULA**				x
**35-R PRECUNEUS**			x	x
**36-R S PARIETAL**				x
**37-R ANG GYRUS**				
**38-R S MARGINAL**	x			x
**39-R WERNICKE**	x	x	x	x
**40-R POSTCENTRAL**				
**41-R PRECENTRAL**				
**42-R FEF**				
**43-R PREMOTOR**	x	x	x	x
**44-R V PREMOTOR**				
**45-R D PREMOTOR**				
**46-R MFG**				
**47-R A MFG**	x	x	x	x
**48-R IF ORBITAL**				
**49-R BROCA**	x	x		
**50-R TRI BROCA**			x	x
**51-R OP BROCA**			x	x
